# *F. Nucleatum* enhances oral squamous cell carcinoma proliferation via E-cadherin/β-Catenin pathway

**DOI:** 10.1186/s12903-024-04252-3

**Published:** 2024-05-02

**Authors:** Zhengrui Li, Yuan Liu, Xufeng Huang, Qi Wang, Rao Fu, Xutao Wen, Ji’an Liu, Ling Zhang

**Affiliations:** 1 Department of Oral and Maxillofacial-Head and Neck Oncology, Shanghai Ninth People’s Hospital, Shanghai Jiao Tong University School of Medicine, No. 639, Zhizaoju Road, Huangpu District, Shanghai, 200011 China; 2 College of Stomatology, Shanghai Jiao Tong University, Shanghai, China; 3 National Center for Stomatology, Shanghai, China; 4 National Clinical Research Center for Oral Diseases, Shanghai, China; 5 Shanghai Key Laboratory of Stomatology, Shanghai, China; 6 Shanghai Research Institute of Stomatology, Shanghai, China; 7 Shanghai Center of Head and Neck Oncology Clinical and Translational Science, Shanghai, China; 8 Faculty of Dentistry, University of Debrecen, Debrecen, Hungary; 9 Jiangsu University, Zhenjiang, China

**Keywords:** *Fusobacterium nucleatum*, Oral squamous cell carcinoma, Cell proliferation, CDH1(E-Cadherin), β-catenin, Phosphorylation, Cell cycle

## Abstract

**Background:**

*Fusobacterium nucleatum* (*F. nucleatum*) is a microbial risk factor whose presence increases the risk of oral squamous cell carcinoma (OSCC) progression. However, whether it can promote the proliferation of OSCC cells remains unknown.

**Methods:**

In this study, we investigated *F. nucleatum* effect on OSCC cell proliferation using in vitro and in vivo experiments.

**Results:**

Our results showed that *F. nucleatum* promoted OSCC cell proliferation, doubling the cell count after 72 h (CCK-8 assay). Cell cycle analysis revealed G2/M phase arrest. *F. nucleatum* interaction with CDH1 triggered phosphorylation, upregulating downstream protein β-catenin and activating cyclinD1 and Myc. Notably, *F. nucleatum* did not affect noncancerous cells, unrelated to CDH1 expression levels in CAL27 cells. Overexpression of phosphorylated CDH1 in 293T cells did not upregulate β-catenin and cycle-related genes. In vivo BALB/c nude experiments showed increased tumor volume and Ki-67 proliferation index after *F. nucleatum* intervention.

**Conclusion:**

Our study suggests that *F. nucleatum* promotes OSCC cell proliferation through the CDH1/β-catenin pathway, advancing our understanding of its role in OSCC progression and highlighting its potential as a therapeutic target.

**Supplementary Information:**

The online version contains supplementary material available at 10.1186/s12903-024-04252-3.

## Introduction

Oral squamous cell carcinoma (OSCC) ranks prominently among head and neck cancers (HNC), characterized by a 5-year survival rate of less than 50% and a pronounced diminishment in the quality of life [1, 2]. Comprehensive understanding of OSCC microbiome, involving multiple molecular interactions, is crucial for its prevention and treatment [3–5]. The microbiome play an essential role in various diseases [3, 6, 7], including cancer. For example, *Helicobacter pylori (H. pylori)* infection leads to gastric ulcers [8, 9], with about part of infected individuals developing gastric cancer. The oral cavity, abundant in microorganisms, maintains oral and overall health through homeostasis [10]. Microorganisms are also linked to OSCC [4, 11], OSCC is associated with carriage of many oral bacteria (such as *Porphyromonas gingivalis (P. gingivalis)*, *Fusobacterium nucleatum (F. nucleatum)*, *Streptococcus*), certain viruses (human papillomavirus (HPV), human herpesvirus 8 (HHV-8), herpes simplex virus 1 (HSV-1), Epstein-Barr virus (EBV) and etc.), and yeast (*Candida albicans*). In addition, members of the oral microbiome have been associated with esophageal, gastric, pancreatic, colon/rectal, and lung cancers [4].

*F. nucleatum*, a gram-negative anaerobic bacillus, is a natural microbiome constituent [12]. Under certain conditions, it can cause opportunistic infections like abscesses, osteomyelitis, and pericarditis [13]. *F. nucleatum* has been shown to promote colorectal cancer development, suppress anti-tumor immunity, and increase chemotherapy resistance [14]. Antibiotic treatment in mice disrupted tumor growth in *F. nucleatum*-bearing colorectal cancer xenografts [15]. *F. nucleatum* abundance increases in OSCC lesions [16], similar to colorectal cancer. Recent studies found that *F. nucleatum* causes DNA damage, promotes OSCC cell proliferation via the Ku70/p53 pathway [17], and induces cisplatin resistance and increased migration by upregulating NFATc3 expression in co-cultured OSCC cells [18]. E-cadherin (CDH1) is crucial for epithelial cell adhesion, and its dysfunction contributes to cancer progression [19]. Reduced CDH1 expression is a hallmark of epithelial-mesenchymal transition (EMT), often associated with cancer progression [20]. CDH1 and β-catenin form a complex that regulates downstream target genes like Cyclin D1 and Myc [21], with its dysregulation linked to poor clinical outcomes in malignant tumors. *F. nucleatum* interacts with CDH1 in colorectal cancer cells [22], promoting tumor progression. Clinical studies have associated CDH1 expression with OSCC prognosis, and low expression indicates poor prognosis and higher metastasis likelihood [23].

Given the significant presence of *Fusobacterium nucleatum* (*F. nucleatum)* in oral squamous cell carcinoma (OSCC) lesions, our research aims to explore the intricate interaction between this bacterium and OSCC cells, particularly focusing on the role of E-cadherin (CDH1). We aim to investigate the influence of *F. nucleatum* on CDH1 phosphorylation and its consequent effects on the CDH1/β-catenin signaling pathway in OSCC. This study strives to enhance our understanding of the molecular mechanisms driving the onset and progression of OSCC, with an aim to contribute to the development of more effective prevention strategies. Additionally, we seek to deepen our knowledge of the complex interplay between microorganisms and cancerous processes. This novel approach could lead to groundbreaking developments in cancer research, potentially transforming both preventive and therapeutic approaches. Our research may also pave the way for improved OSCC screening methods, increasing the accuracy of early diagnoses, and could lead to the development of new therapeutic strategies targeting F. nucleatum and its related signaling pathways, offering promising additions to current OSCC treatments.

## Methods

### Cells and bacterial culture

The cell lines used in this study (CAL27, 293T, HGF, and HOK cells) were obtained from the Shanghai Key Laboratory of Stomatology and were cultured in DMEM (11,965,092, Gibco, US) or RPMI 1640 (22,400,071, Gibco) supplemented with 10% (v/v) fetal calf serum (26,010,074, Gibco) and 1% (v/v) penicillin G/streptomycin at 37 °C with 5% CO2 (15,240,062, Gibco, ). *F. nucleatum* (25,586, ATCC, US) was cultured in brain heart infusion (BHI) medium (53,283, Sigma-, US) in an anaerobic chamber (Baker Ruskinn Bugbox Plus, UK) with an atmosphere of 90% N2, 5% CO2, and 5% H2 at 37 °C. *S. mutans* (25,175, ATCC) was cultured with BHI medium and grown in an aerobic chamber. Before the experiment, the bacteria were sterilized at 75 °C for 5 min and centrifuged at 4000 g for 20 min, and the supernatant was aspirated. After sterilization, microscopic observation of the bacteria was performed to ensure they remained morphologically intact. The supernatant was centrifuged at 14,000 g for 20 min to obtain the supernatant used in the experiment.

The multiplicity of infection (MOI, The ratio of the number of infection units (such as bacterial cells) to the number of host cells) of *F. nucleatum* (*Fn*) at 500 was chosen based on preliminary dose-response experiments. We observed that at this MOI, the bacterial cells were efficiently internalized by the cancer cells without causing significant cell death. Our extensive pre-experimental mapping in the early stages revealed this, especially the MOI ratio, which is different from the 1:100 of most studies, with around 1:500 to harvest the best proliferation-promoting effect.

### Detection of cell proliferation

Cells were seeded in hexaplicate at a density of 3000 cells/well in 96-well cell culture plates and incubated for three days, with or without *F. nucleatum* at a multiplicity of infection (MOI) of 500:1. Cell proliferation was evaluated using the Cell Counting Kit-8 (CCK-8) (CK04 Dojindo Japan), and the OD values at 450 nm were measured using a microplate reader (Tecan Infinite 200 PRO) at the same time point each day. Cell counts were also determined in triplicate in 24-well plates seeded with 1 × 10^4^ cells per well and counted at 24-hour intervals using a hemocytometer. Each experiment was repeated at least three times.

### Plate clone formation assay

Cells were collected and resuspended to obtain a single-cell suspension, which was then seeded in 35 mm cell culture dishes at 200 cells/dish density. After 24 h, *F. nucleatum* was added at a multiplicity of infection (MOI) of 500, and the medium was changed every 2–3 days with half of the medium being replaced to avoid disturbing the cell colonies and bacterial abundance. After approximately 14 days, the number of cells in each clone was counted under a microscope, and clones with more than 20 cells were fixed with methanol and stained with Wright-Giemsa stain (Baso, China).

### Cell cycle assay

The cells were seeded in 6-well plates and co-cultured with *F. nucleatum* at an MOI of 500. The cells were fixed with ice-cold 70% ethanol for cell cycle analysis and incubated at 4 °C for 24 h. Before analysis, the cells were washed with PBS and stained with propidium iodide (PI, 10ug/ml)(40710ES03, Yeasen, China) in the dark for 30 min. The cell cycle was then analyzed using a flow cytometer (BD LSRFortessa, US).

### Real-time PCR

RNA was extracted from cells using TRIzol, and cDNA was synthesized using the PrimeScript RT Reagent Kit (TaKaRa Japan). Quantitative PCR (qPCR) was performed using FastStart Universal SYBR Green Master Mix (ROX) (Roche, Switzerland) with the Roche LightCycler 480 II Real-Time PCR system (Roche Switzerland). All genes were measured in triplicate, and primers were purchased from Sangon Biotech (Shanghai, China). The primers used for Myc were 5′- CCTAGTGCTGCATGAGGAGA-3′ (forward) and 5′- TCTTCCTCATCTTCTTGCTCTTCG-3′ (reverse); primers for Cyclin D1 were 5′- TGCCCTCTGTGCCACAGATG-3′ (forward) and 5′- TCTGGAGAGGAAGCGTGTGAG-3′ (reverse), and primers for GAPDH were: 5′- TGCACCACCAACTGCTTAG-3′ (forward) and 5′- GATGCAGGGATGATGTTC-3′ (reverse). The GAPDH mRNA level was used for normalization, and data were analyzed using the 2^-ΔΔCT method.

### Western blot

Cells were washed with ice-cold PBS and lysed using a Membrane and Cytosol Protein Extraction Kit (20127ES60, Yeasen) containing protease (20124ES03, Yeasen) and phosphatase inhibitors (20109ES05, Yeasen). After collecting the cell samples, centrifuge at 700 g for 10 min at 4℃, transfer the supernatant to a new EP tube, do not touch the precipitate. Centrifuge at 14,000 g for 30 min at 4℃, precipitate the cell membrane fragments, transfer the supernatant to a new EP tube, and obtain the cell plasma protein. The above precipitate was centrifuged at 14,000 g for 10s at 4 °C and the supernatant was aspirated as much as possible. Then add 200 µL of B solution (BCA Protein Assay Kit, containing PMSF) to the precipitate, vortex for 5 s, ice bath for 5 min, and repeat 2 times. Then centrifuge at 14,000 g for 5 min at 4 °C. The supernatant is the membrane protein. The protein concentration was determined using the BCA Protein Assay Kit (20201ES76, Yeasen) as per the manufacturer’s protocol. Total proteins were resolved on 4–20% Bis-Tris gels (Genscript China) and transferred onto PVDF membranes (Millipore ISEQ00010). Subsequently, the membranes were blocked with 5% skim milk in TBST(Tris-Buffered Saline Tween) at room temperature for 1 h. The following primary antibodies were diluted in 0.5% skim milk in TBST and incubated with the membrane at 4 °C overnight: anti-EGFR (EGFR, a typical transmembrane protein gene, was used as a control)(Cell Signaling Technology CST #4267, 1:1000 dilution), anti-CDH1 (Absin, abs136677, 1:1000 dilution), anti-phosphorylated CDH1 (Absin abs140194, 1:1000 dilution), anti-Cyclin D1 (CST #55,506, 1:1000 dilution), anti-β-catenin (CST #8480, 1:1000 dilution), anti-β-actin (Absin abs830031, 1:1000 dilution), and anti-Myc (Absin abs115533, 1:1000 dilution). Following three washes with TBST, the membrane was incubated with an HRP-conjugated secondary antibody (34201ES60 and 34101ES60, Yeasen) in TBST at room temperature for 1 h. The immunoreactive bands were detected using ECL(Enhanced Chemiluminescence) Western Blotting Substrate (36208ES60, Yeasen), and band density was quantified by Image J. All full-length blots/gels are presented in Supplementary files.

### Transient transfection experiment

The plasmid and siRNA were constructed by Genomeditech (Shanghai, China). The CDH1 plasmid was transfected into cells using the DharmaFECT kb transfection reagent (#13,778,075, Thermo Scientific, US), and siRNAs were transfected using Lipofectamine 2000 (Thermo Scientific, US) according to the manufacturer’s instructions. The *β-cat*enin (h)-si RNA sequences were 5′-GGGUAGGGUAAAUCAGUAA-3′ and 5′-UUACUGAUUUACCCUACCC-3′, and the CDH1 plasmid sequence is shown in the supplemental file (F: 5′-AATCCAAAGCCTCAGGTCATAAACA-3′; R: 5′-GGTTGGGTCGTTGTACTGAATGGT-3′).

### Establishment of a subcutaneous xenograft model in nude mice

A total of 16, four-week-old wild-types BALB/c nude mice (8 males and 8 females) were obtained from Shanghai SLAC Laboratory (Shanghai, China) and housed under standard laboratory conditions at the NHC Key Lab of Reproduction Regulation. The mice were randomly divided into two groups, and a back subcutaneous injection of a 100 µL solution containing 1*10^6 CAL27 cells was administered. *F. nucleatum* was injected once a week for 3 weeks by using sterile fine needle syringe (bacterial solution for injection is transferred in advance using transferpette), while PBS was used as a control, with the injection volume set at 50 µL of *F. nucleatum* in PBS, and an absorbance of 1 at 600 nm(1*10^8 *F. nucleatum*). Tumor volumes were measured weekly and calculated using the formula: length × (width)^2 × 1/2. The animal experiments were conducted following a protocol approved by the NHC Key Lab of Reproduction Regulation (ethical approval number 2020-32).

### Immunohistochemistry

The tumor tissue was immediately fixed in 10% buffered formalin and embedded in paraffin. Tumor Sect. (4 µm) were deparaffinized and rehydrated in a graded ethanol series and distilled water. For antigen retrieval, sections were immersed in 0.01 M sodium citrate buffer and heated in a steamer for 30 min. After incubation with 3% hydrogen peroxide to inhibit endogenous peroxidase activity and blocking with goat serum albumin, the sections were incubated with anti-CDH1 (Abcam ab40772, 1:200), anti-p-CDH1 (Abcam ab76319, 1:200), and anti-Ki67 antibodies (Abcam ab15580, 1:200) overnight at 4°C. Subsequently, sections were incubated with an HRP-labeled secondary antibody (34201ES60, Yeasen), and then stained with 3,3’-diaminobenzidine tetrahydrochloride (DAB) for 5 min. Protein expression was quantified using Image-Pro Plus software 6.0 from Media Cybernetics (Rockville, US).

### Statistical analyses

GraphPad Prism software version 8.0 was used for statistical analyses. Statistical tests used are indicated in the figure legends. Student’s t-test and one-way ANOVA were used to analyze the significance of differences. Wilcoxon rank-sum test was carried out to compare the difference between groups. All *P* values were two-tailed, and *P* values less than 0.05 were considered statistically significant.

## Result

### *F. nucleatum* promoted OSCC cells proliferation

**Fig. 1:**
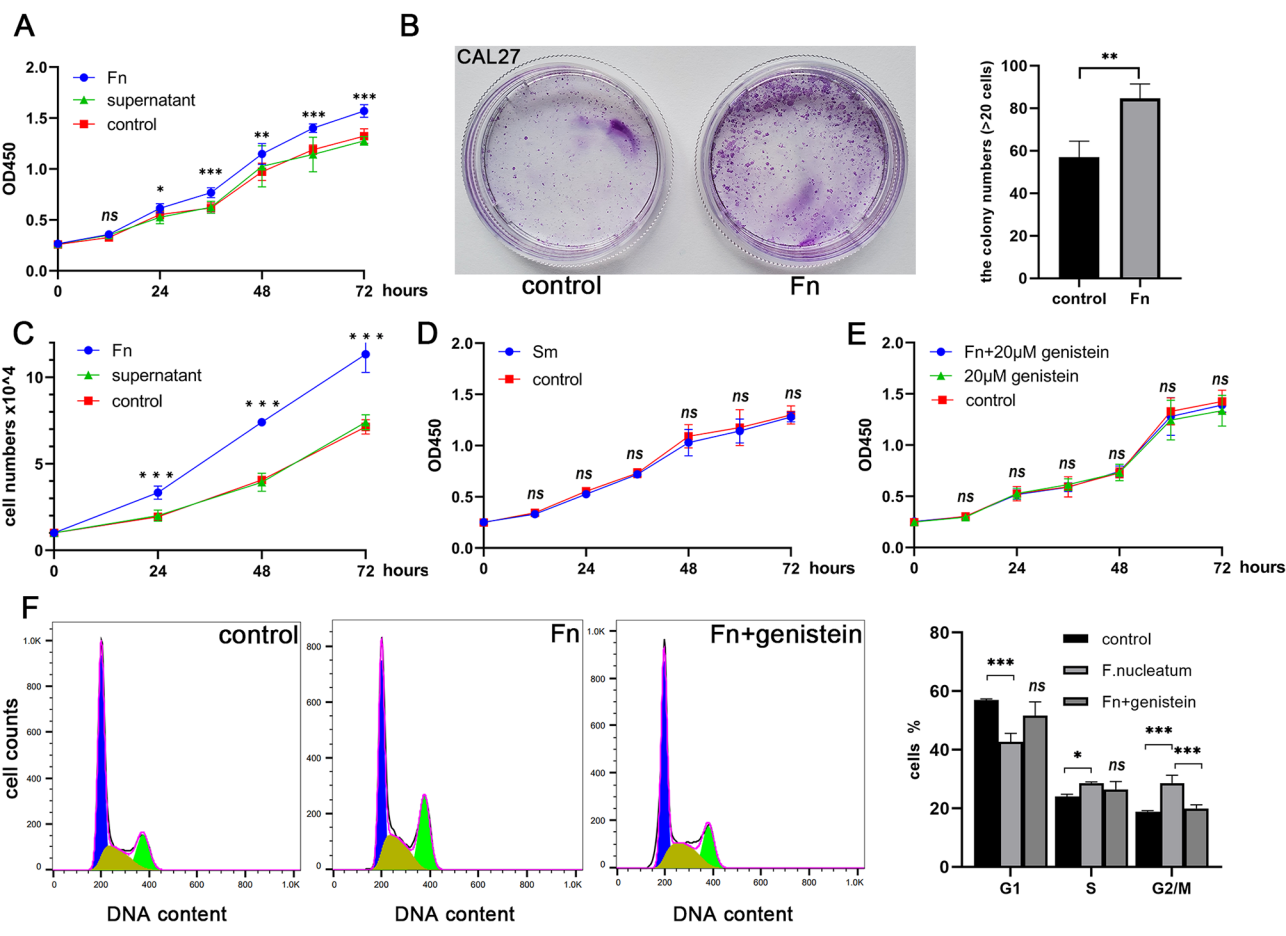
Methodological approach for studying cell proliferation in OSCC cells, with a focus on CAL27, conducted in three replicates. (**A**) Utilizing the CCK-8 assay, the influence of *F. nucleatum* and its supernatant on CAL27 cell proliferation was assessed. (**B**) Method to determine cell clone formation post-*F. nucleatum* intervention. (**C**) A 72-hour cocultivation followed by a cell counting procedure revealed an approximately 100% acceleration in cell growth, though the supernatant displayed negligible impact. (**D**) Control observation was employed to assess the proliferation effects of S. mutans on the cells, indicating no induction of cell proliferation. (**E**) Post-exposure effects of genistein on CAL27 cells in relation to (**F**) *nucleatum* was analyzed, highlighting genistein’s nullifying effects on *F. nucleatum*’s pro-proliferative attributes. F. A comparative cell cycle analysis encompassing the control, the *F. nucleatum*-treated, and the genistein-treated groups was performed, accompanied by statistical evaluations. (*, *P*<0.05; **, *P*<0.01; ***, *P*<0.001.)

The oral tongue cancer cell line CAL27 was co-cultured with *F. nucleatum* at an MOI of 500, resulting in a significant increase in the absorbance value of CCK-8, indicating that *F. nucleatum* stimulated the proliferation of CAL27 cells. This effect was not observed with bacterial culture supernatant (Fig. 1A). In addition, *F. nucleatum* stimulation led to the formation of more cell clones in the plate cloning assay, and this finding was confirmed in independent experiments performed in triplicate (Fig. 1B). To confirm the effect of *F. nucleatum* on cell growth, a cell count experiment was conducted in a 24-well plate, which showed a ∼ 100% increase in CAL27 cell growth compared to untreated cells after 72 h. The bacterial supernatant did not affect the cell count (Fig. 1C). Furthermore, unlike *F. nucleatum*, Streptococcus mutans (S. mutans) did not promote OSCC cell proliferation (Fig. 1D). When the phosphorylation inhibitor genistein was added, the cell proliferation curve did not change significantly under *F. nucleatum* coculture conditions (Fig. 1E). Flow cytometry analysis revealed that *F. nucleatum* treatment increased the percentage of cells in the G2/M phase by 10%, consistent with *F. nucleatum* promoting cancer cell proliferation (Fig. 1F). Moreover, there was a slight increase in the number of cells in the S phase, suggesting a broader impact of F. nucleatum on the cell cycle phases, potentially through specific signaling pathways or molecular interactions that warrant further investigation.

### The phosphorylated CDH1 protein played a key role in the proliferation

CDH1, a transmembrane protein, is closely associated with cancer cell proliferation and metastasis. Upon coculture with F. nucleatum, a significant modification was observed in the behavior of the CDH1 protein. Notably, phosphorylation of CDH1 was induced, leading to a marked reduction in its presence on the cell membrane. However, it’s important to note that the total level of CDH1 in the cell remained constant. This phosphorylation event had profound downstream effects. The phosphorylated CDH1 was translocated from the membrane to the cytoplasm, a shift that is critical for understanding its role in cancer progression. Once in the cytoplasm, phosphorylated CDH1 engaged in interactions with various pathway proteins, altering their functions and activities.

**Fig. 2:**
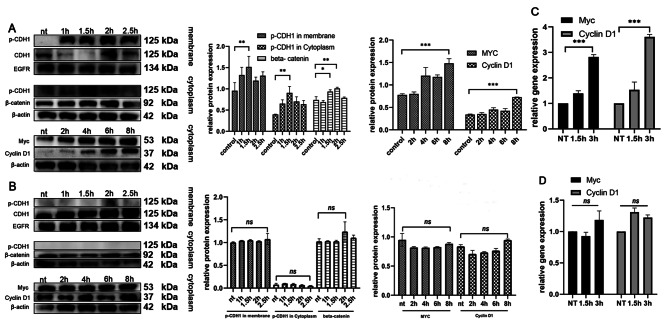
Analytical procedures and findings pertaining to CDH1 expression and its downstream molecules following diverse treatments, utilizing Western blot and qPCR techniques. (**A**) Western blot (WB) was employed to monitor the modulation of CDH1 and its downstream proteins after *F. nucleatum* exposure. Notably, there was an upregulation in the cytoplasmic protein β-catenin, subsequently elevating the levels of Myc and Cyclin D1. (**B**) Post-genistein treatment analysis revealed an inhibition in CDH1 phosphorylation, ensuring stable expression of CDH1 and its downstream entities. (**C**) For oncogene expression post *F. nucleatum* treatment, qPCR was the chosen technique. This assessment highlighted an elevation in oncogene expression after *F. nucleatum* exposure. (**D**) Following genistein administration, qPCR outcomes indicated no marked disparities in oncogene expression levels. (*, *P*<0.05; **, *P*<0.01; ***, *P*<0.001.)

One of the most significant consequences of this translocation and interaction was the increased expression of β-catenin, a molecule well-known for its cancer-promoting properties. This upregulation was accompanied by an increase in the levels of oncogenes Myc and Cyclin D1 (Fig. 2A and C). These changes point to a heightened state of cellular proliferation and a potential increase in metastatic capabilities. Interestingly, in terms of cell cycle analysis, no marked changes were observed, suggesting that the influence of phosphorylated CDH1 on the cell cycle might be subtle or occur through indirect pathways.

Furthermore, with prolonged coculture times, there was a noticeable decrease in the level of phosphorylated CDH1. This reduction correlated with a stabilization in the level of the downstream protein β-catenin, indicating a dynamic interaction between these molecules over time. Additionally, the expression levels of Myc and Cyclin D1 did not show significant variation after this period (Fig. 2B and D). This observation suggests a temporal aspect to F. nucleatum’s influence on CDH1 phosphorylation and its downstream effects, potentially indicating a complex regulatory mechanism at play in the cancer cell response to bacterial interaction.

### Knockdown of β-catenin inhibited downstream oncogene expression

In our study, we conducted targeted knockdown and overexpression experiments to elucidate the specific role of β-catenin in F. nucleatum-mediated OSCC cell proliferation, as illustrated in Figure S1. A key aspect of these experiments was the knockdown of β-catenin. When β-catenin levels were reduced, we observed a significant downregulation in the expression of downstream oncogenes. This finding is pivotal as it underscores the critical role of β-catenin as a mediator in the signaling pathway leading to cell proliferation. Notably, despite the reduction in oncogene expression, the levels of upstream proteins remained unchanged. This indicates that β-catenin acts downstream of these proteins and is a key regulator of oncogene expression.

**Fig. 3:**
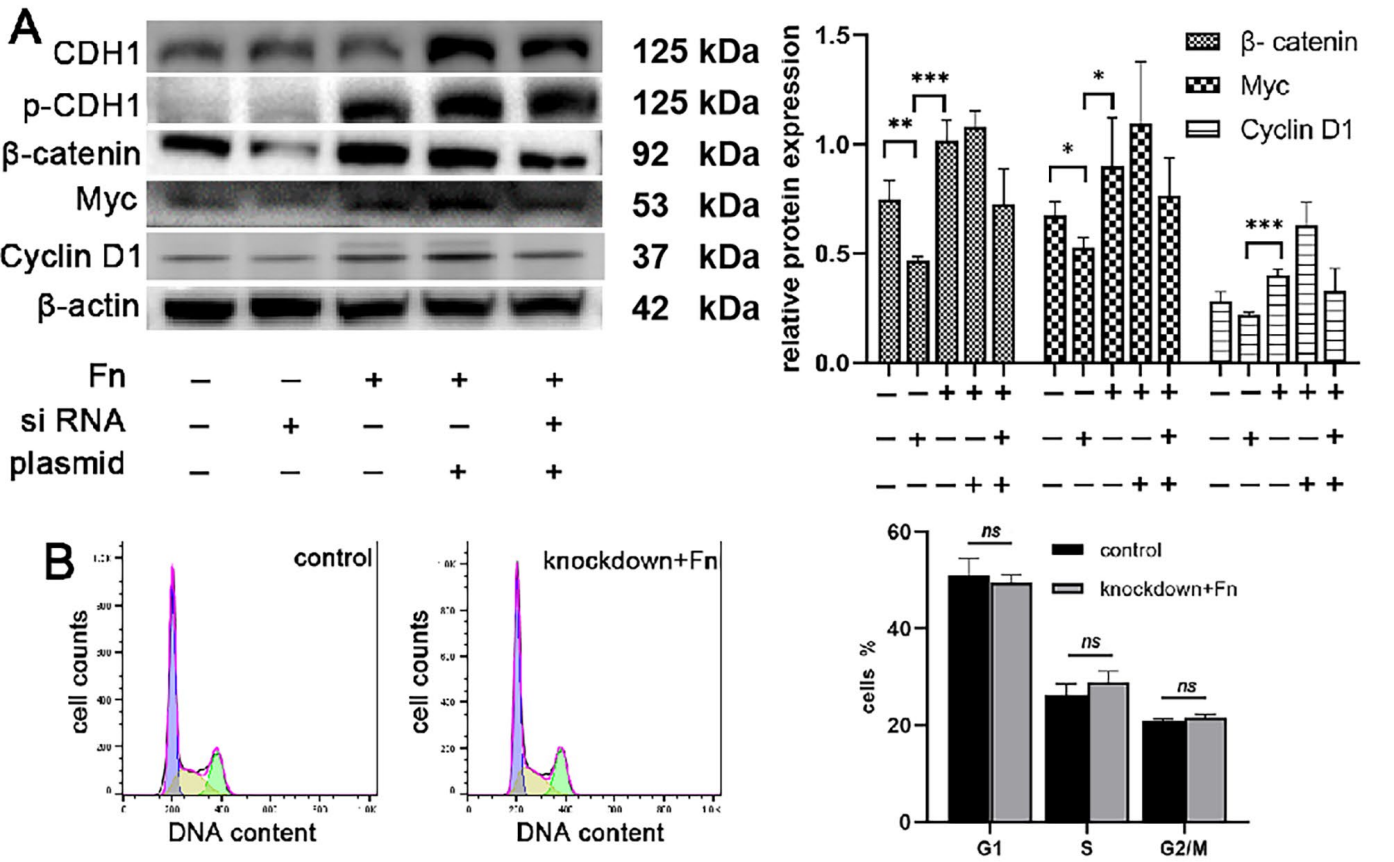
The effect of CDH1/β-catenin on cell proliferation. (**A**) Changes in the expression of CDH1 and its downstream proteins after transient transfection. After knocking down β-catenin, downstream protein expression was inhibited. After CDH1 overexpression, oncogene expression increased slightly. When cells overexpressed CDH1 and β-catenin were silenced simultaneously, the expression of downstream Myc and Cyclin D1 decreased, indicating that β-catenin was the key protein that activated downstream signaling. (**B**) Cell cycle analysis following knockdown β-catenin, the cell cycle was not different from the control group

Furthermore, to dissect the interaction between CDH1 and β-catenin, we conducted experiments where CDH1 was overexpressed while β-catenin was simultaneously knocked down. Interestingly, under these conditions, there was no significant change in downstream oncogene expression, despite the observed increase in both CDH1 and phosphorylated CDH1 (p-CDH1) levels (Fig. 3A). This result suggests that while CDH1 and its phosphorylated form may play a role in this pathway, β-catenin is an essential component for the activation of downstream signaling cascades that promote cell proliferation.

Adding another layer of complexity to our findings, flow cytometry analysis was performed to assess the impact of β-catenin knockdown on the cell cycle, especially in cells treated with F. nucleatum. Interestingly, even with F. nucleatum intervention, the G2/M ratio did not show a considerable increase following β-catenin knockdown (Fig. 3B). This observation further cements the critical role of β-catenin in F. nucleatum-induced OSCC cell proliferation. It implies that β-catenin is not only crucial for driving oncogene expression but also plays a pivotal role in advancing the cell cycle, particularly in transitioning to the G2/M phase, a key phase associated with cell proliferation and growth.

### *F. nucleatum* selectively promoted OSCC cell proliferation

**Fig. 4:**
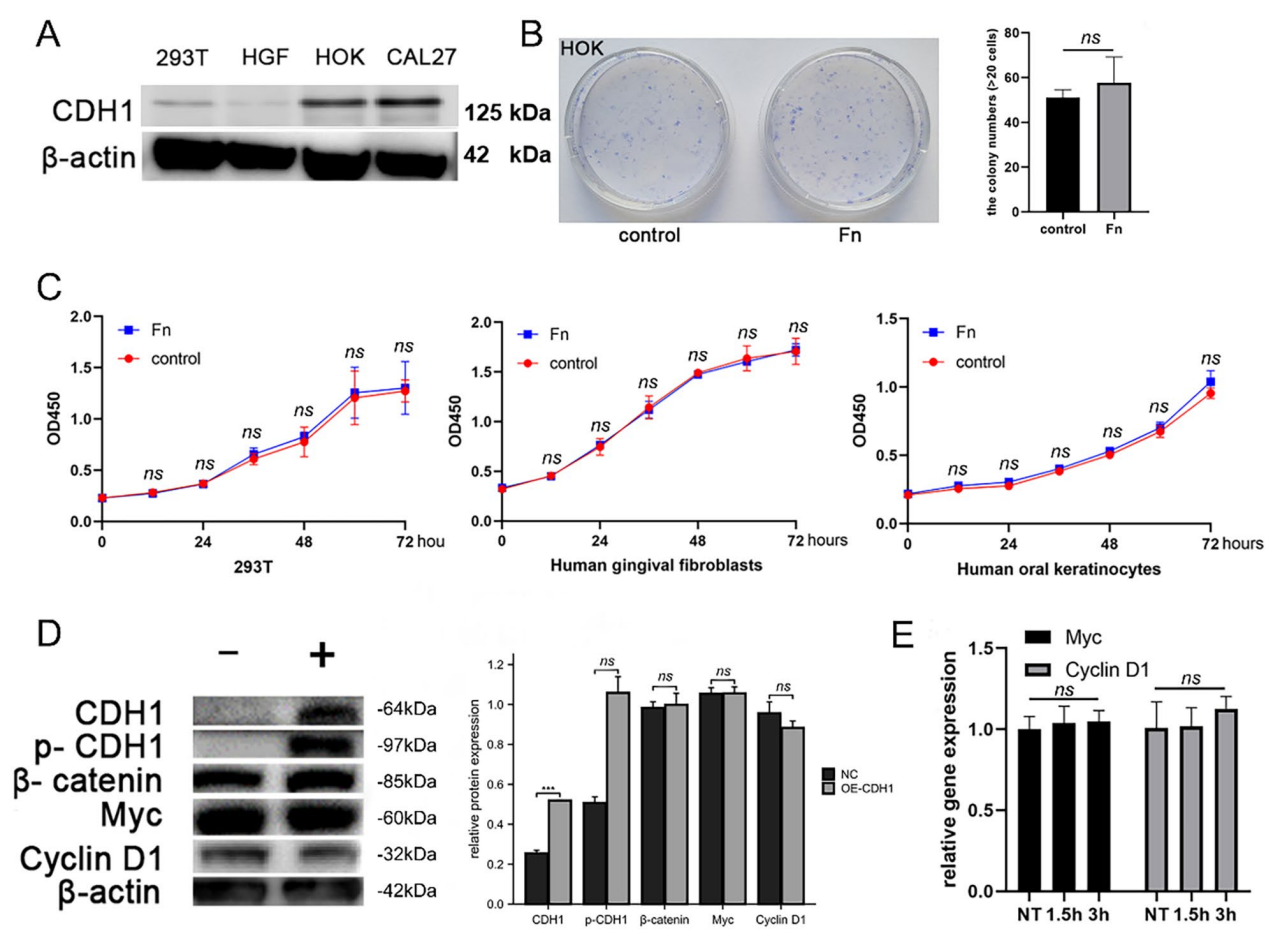
Detection of *F. nucleatum* on the proliferation of noncancerous cells. (**A**) Detection of CDH1 expression in four cell lines revealed differential expression in tumor cell lines, human oral keratinocytes (HOK), 293T cells, and human gingival fibroblasts (HGF). (**B**) After *F. nucleatum* was cocultured with 293T, HGF and HOK cells, the CCK-8 assay showed no obvious promotion of proliferation. (**C**) There was no difference in the plate clone formation observation after culturing *F. nucleatum* with HOK cells present. (**D**) In 293T cells overexpressing CDH1, phosphorylated CDH1 was detected, but downstream signaling was not activated. (**E**) No difference in oncogene expression was detected using qPCR in 293T cells overexpressing CDH1 and stimulated with (**F**) *nucleatum*

CDH1 expression in CAL27 cells was found to play a key role in promoting proliferation. However, it was unclear whether *F. nucleatum* also promoted the proliferation of noncancerous cells. Before investigating whether *F. nucleatum* altered the proliferation of noncancerous cells, the expression of the CDH1 protein was first detected in human gingival fibroblast (HGF), 293T cells, human oral keratinocyte (HOK), and CAL27 cells. HGF and 293T cells showed no or very little expression of CDH1. In contrast, HOKs and CAL27 cells expressed relatively higher levels of CDH1 (Fig. 4A). Under coculture conditions, *F. nucleatum* did not promote the proliferation of HGF and 293T cells (Fig. 4B). Although CDH1 was expressed at high levels in HOK cells, the plate cloning experiment confirmed that *F. nucleatum* did not affect the proliferation of HOK (Fig. 4C). In 293T cells transfected with the CDH1 plasmid, CDH1 and p-CDH1 levels increased under coculture conditions, but β-catenin levels did not increase. The downstream target genes were stably expressed (Fig. 4D and E). Thus, although CDH1 was expressed at high levels in noncancerous cells, *F. nucleatum* did not promote their proliferation, suggesting that certain changes specific to cancer cells are required for *F. nucleatum* to promote proliferation.

### In vivo experiment to verify the ability of *F. nucleatum* to promote tumor growth

**Fig. 5:**
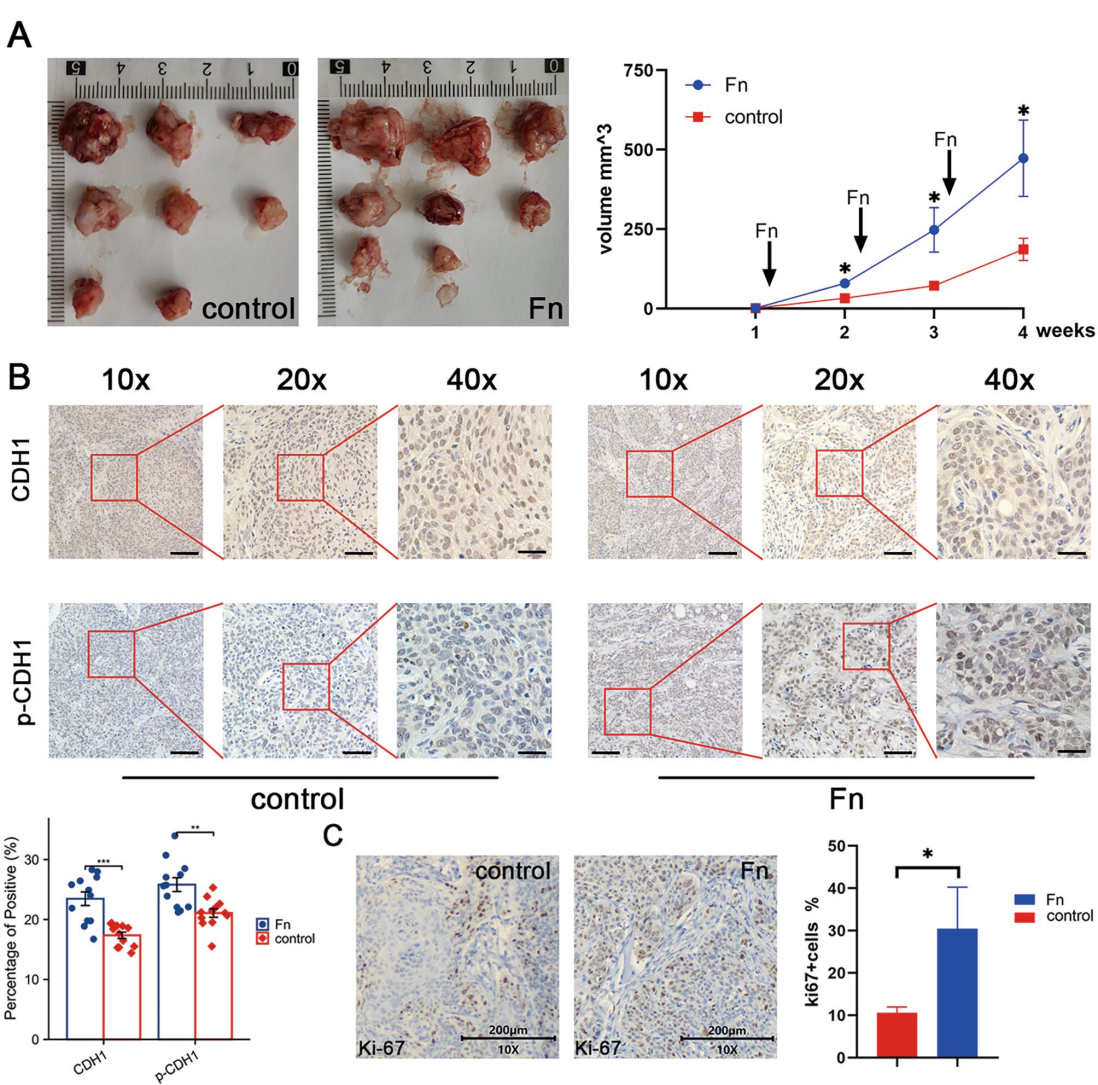
Xenografts in nude mice. (**A**) After the *F. nucleatum* injection, nude mice had larger tumors, and the difference was obvious. (**B**) IHC showed the changes in CDH1 and p-CDH1 expression at different magnifications. CDH1 was expressed in both the control group and the *F. nucleatum* group. Because this protein is mainly located in the cell membrane, IHC revealed a diffuse expression pattern. The fibroblasts in the tumor were not stained, indicating that they did not express CDH1. IHC showed obvious increases in p-CDH1 expression in the *F. nucleatum* intervention group. Compared with the control group, the protein was upregulated and appeared in the cytoplasm. Scale bars, 80, 40 and 20 μm at 10x, 20x and 40x magnification, respectively. (**C**) The IHC analysis of changes in Ki-67 expression showed a higher positive rate in the *F. nucleatum* intervention group

CAL27 cells were inoculated into nude mice, followed by three injections of either PBS or inactivated bacteria to examine the effect of *F. nucleatum* on OSCC growth in vivo. Tumor growth was increased after four weeks of treatment with *F. nucleatum* compared to animals treated with PBS (Fig. 5A). Using immunohistochemistry (IHC), we analyzed the changes in CDH1 and p-CDH1 levels. CDH1 was originally expressed in both the control and *F. nucleatum* groups. The positive cells were light brown. Because E-cadherin protein is mainly located in the cell membrane, a diffuse expression pattern was observed. The fibroblasts in the tumor were not stained, indicating no CDH1 expression, and the cells were light blue. When IHC showed changes in p-CDH1 expression, the *F. nucleatum* intervention group showed an obvious increase in p-CDH1 levels. Compared with the control group, the E-cadherin protein was upregulated and appeared in the cytoplasm (Fig. 5B). IHC staining using anti-Ki67 antibodies was performed to determine cancer cell proliferation status. The *F. nucleatum*-treated mice showed a higher ratio of Ki-67 positivity (Fig. 5C).

## Discussion

During routine oral examinations, clinicians often observe a correlation between suboptimal oral hygiene and compromised oral health. A myriad of oral diseases, encompassing oral squamous cell carcinoma (OSCC), exhibit strong associations with the oral microbiota [4, 7, 16]. For instance, *Streptococcus mutans (S. mutans)* has been implicated in dental caries [11], while *Porphyromonas gingivalis* (*P. gingivalis*) has been linked to periodontal disease [24]. The distinct anatomical structure of the oral cavity promotes the growth of a diverse microbial community. Previous research has identified a connection between insufficient oral hygiene and oral cancer [16]. To investigate this relationship, experimental studies have utilized chemical approaches to induce OSCC in murine models [25], followed by transplantation of the OSCC-associated microbiome into the oral cavities of mice upon successful model establishment [26]. The findings revealed a significant increase in tumor number and volume compared to control groups, thus highlighting the potential impact of oral microbiota on cancer development [26].

The capacity of *F. nucleatum* to serve as a bridging organism among distinct bacterial species and its facilitation of multispecies biofilm formation accentuates its significance as an opportunistic oral pathogen [12]. While earlier research primarily focused on the involvement of *F. nucleatum* in periodontal disease, recent investigations have elucidated its potential role in cancer pathogenesis. The invasion of oral epithelial cells by *F. nucleatum* activates NF-κB and triggers the production of proinflammatory cytokines, consequently resulting in alveolar bone resorption and periodontitis induction [27]. Substantial microbiota variations have been observed across diverse anatomical regions within the oral cavity. Notably, studies have identified a markedly higher abundance of *F. nucleatum* within the microbiota of OSCC compared to other oral sites [28, 29], highlighting its possible association with OSCC. A more comprehensive understanding of the role of *F. nucleatum* in cancer pathogenesis could pave the way for the development of innovative preventative and therapeutic strategies targeting OSCC [30]. Furthermore, *F. nucleatum*’s role in OSCC is linked to its ability to create an immunosuppressive Tumor Microenvironment (TME) [31]. It inhibits immune cell activity and promotes a pro-inflammatory and oncogenic environment, aiding tumor growth and evasion from immune surveillance. This aspect of *F. nucleatum*’s interaction with the TME underscores its potential as a target for therapeutic interventions in OSCC management.

**Fig. 6:**
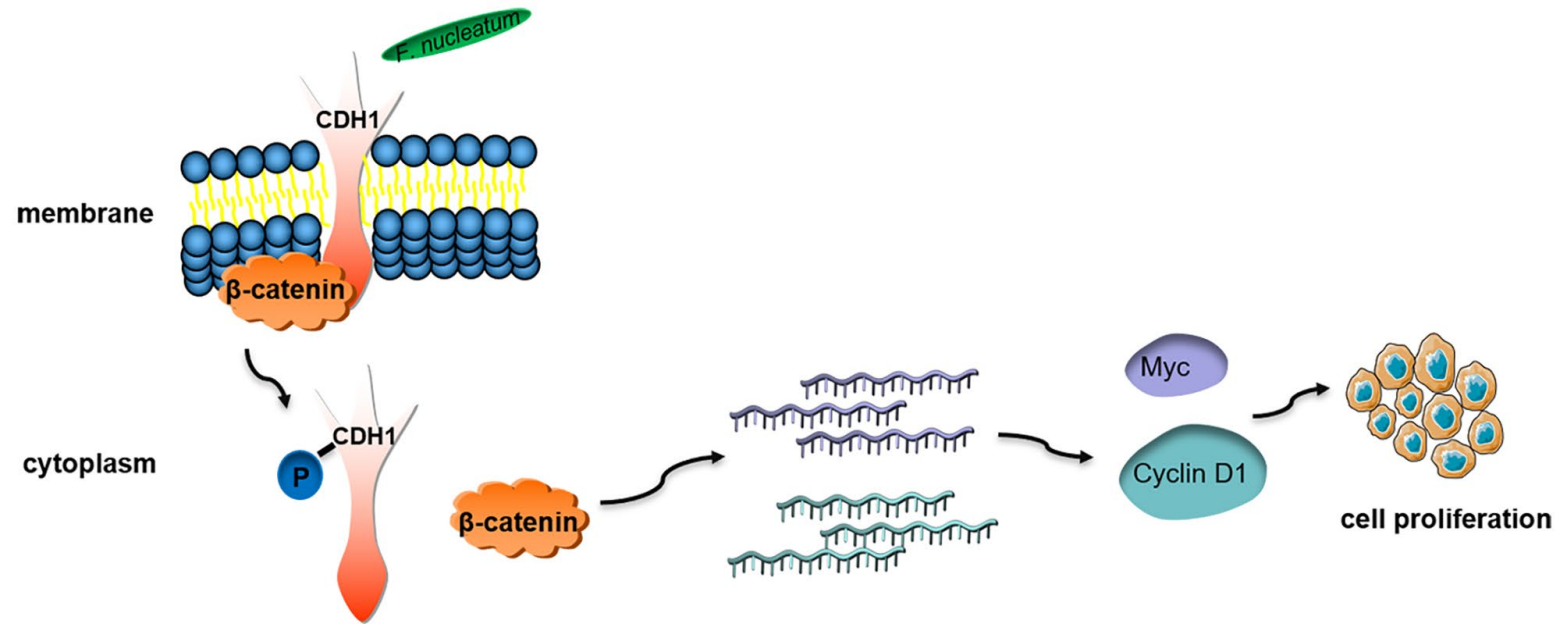
The pattern diagram of *F. nucleatum* promotes OSCC cell proliferation. *F. nucleatum* interacts with CDH1 on the membrane, and CDH1 is phosphorylated and enters the cytoplasm. β-Catenin is isolated and subsequently activates the transcription of downstream oncogenes. And OSCC cell proliferation is increased

Our experimental findings revealed that *F. nucleatum* fostered the proliferation of OSCC cells. Under coculture conditions of *F. nucleatum* and OSCC cell, assessments such as the CCK-8 assay, plate colony formation, cell cycle analysis, and nude mouse tumor xenografts displayed heightened cell proliferation. The CDH1/β-catenin pathway was instrumental in this process. Coculture conditions resulted in the upregulation of β-catenin, Myc, and Cyclin D1, subsequently inducing cell proliferation. The phosphorylation of CDH1 proved essential for this process, as p-CDH1 entered the cytoplasm to interact with downstream proteins and activate signaling pathways. The protein tyrosine kinase (PTK) inhibitor genistein attenuated CDH1-mediated signaling [32]. Experiments demonstrated that p-CDH1 upregulated β-catenin. CDH1 and β-catenin are closely interrelated, without activated CDH1, β-catenin fails to transmit further signals. Upon β-catenin knockdown, downstream oncogenes were downregulated. Cyclin D1, a prevalent oncogene overexpressed in OSCC [33, 34], has strong associations with clinical indicators such as tumor-node-metastasis (TNM) stage, high histological grade, and poor prognosis. The Myc family exhibits amplified expression in various solid malignancies, contributing to tumor growth and drug resistance [35, 36]. Myc is also implicated in malignant transformation. Upregulated β-catenin translocates to the nucleus to activate downstream targets such as T-cell factor (TCF) and lymphoid enhancer factor (LEF), inducing Myc and Cyclin D1 gene transcription [37]. Cyclin D1 and CDK4 form a complex that dissociates from the RB-E2F complex, activating Cyclin D1 [38]. The positive feedback loop among RB, E2F, and Cyclin D1 augments the number of cells in G1/S and S phases. The unrestrained growth of cells arises from the elimination of the requirement for target gene transcription. Based on these observations, we postulate that *F. nucleatum* accelerates the OSCC cell cycle and stimulates cell proliferation (Fig. 6).

The impact of *F. nucleatum* on non-cancerous cells remains ambiguous. To explore this, we investigated human gingival fibroblasts (HGF) and human oral keratinocytes (HOK) cells, which both originate from the oral cavity and display a close association with *F. nucleatum*. Intriguingly, we did not observe any increase in the proliferation of these cells. Given the critical role of CDH1, we transfected 293T cells with the CDH1 plasmid and cocultured them with *F. nucleatum*, which led to elevated p-CDH1 levels. However, downstream signals were not further activated, suggesting that *F. nucleatum* does not engage the CDH1/β-catenin pathway in noncancerous cells. Our findings imply that the influence of *F. nucleatum* on noncancerous cell proliferation is not related to CDH1 expression levels. Our results indicate that *F. nucleatum* does not promote proliferation prior to malignant transformation, regardless of whether epithelial or mesenchymal cells are examined. Several studies have elucidated the direct or indirect role of *F. nucleatum* in colorectal cancer. Fap2, a protein expressed by *F. nucleatum*, facilitates the bacterium’s binding to Gal-GalNAc on cancer cells [13]. In a study employing coculture of *F. nucleatum* and human immortalized oral epithelial cells (HIOECs), the differential expression of 353 mRNAs was observed [39], and the expression of multiple oncogenes, such as CREM, CREB1, and NCOA, was upregulated. *F. nucleatum* also bound and activated the cell inhibitory receptor CEACAM1 on CEACAM1 + tumor-infiltrating lymphocytes and CEACAM1 + tumor cells, indicating its potential significance in modulating antitumor immunity and aiding the tumor in evading immune cell attack through an additive mechanism [25]. Another study cocultured *F. nucleatum* with human gingival fibroblasts (HGF), which activated the AKT/MAPK pathway and induced the expression of inflammatory factors [40]. The authors suggested that *F. nucleatum* did not promote non-oral cancer cell proliferation, which is consistent with the results of our research on noncancerous cells in the oral cavity.

Numerous studies have emphasized the role of E-cadherin as a tumor suppressor [23]. E-cadherin governs Ca2+-dependent adhesion to specific cells and acts as a critical regulator of morphogenesis and signaling pathways in various organs [23]. The cadherin-catenin complex, established by E-cadherin binding to β-catenin, mediates cell adhesion and enhances mechanical stability [41]. CDH1 alterations at both gene and epigenetic levels lead to reduced E-cadherin expression, resulting in decreased cell adhesion and cancer-promoting signals [42]. Dysregulation of the cadherin-catenin complex impairs cell adhesion. Some genetic-level CDH1 alterations include inactivating mutations and loss of heterozygosity (LOH), found in infiltrating lobular breast carcinomas, and heterozygous germline mutations that increase the lifetime risk of developing diffuse gastric cancer and lobular breast cancer. Most commonly, E-cadherin expression is reduced via epigenetic alterations at various levels, including promoter methylation and histone modifications [41]. Dysfunction of the cadherin-catenin complex results in β-catenin accumulation in the cytoplasm and nucleus of tumor cells [43], promoting downstream signaling pathways related to cell proliferation. The function of β-catenin is equally crucial, as its interaction with cadherin connects adhesion junctions to filamentous actin and promotes the reorganization of the actin cytoskeleton [44]. For example, phosphorylation of either E-cadherin or β-catenin affects β-catenin binding to cadherin, disassembling the complex structure. β-Catenin mediates numerous signaling processes linking the cadherin-catenin complex to the cytoskeleton [45]. The complex maintains the balance between cell-cell adhesion and cell differentiation, modulates cell growth and motility, and is vital for the cells’ capacity to adhere to each other [45]. Signaling is activated at the site of the cadherin-catenin interaction. Certain alterations have been observed in cancer cells, inducing the activation of downstream proliferation-related genes, which accelerates cell proliferation. However, this abnormality did not occur in noncancerous cells, and *F. nucleatum* did not promote their proliferation.

Although in vitro cellular assays and animal models can provide preliminary insights into the functional and mechanistic aspects of *F. nucleatum*-mediated CDH1 phosphorylation in regulating the CDH1/β-catenin pathway in OSCC, they cannot fully replicate the human physiological environment. However, a single tumor cell line may limit our conclusions. In future studies, we will consider using other OSCC cell lines to further validate our findings. However, choosing a subcutaneous model over an in situ model does have its limitations. Subsequent studies will be conducted using in situ models that can more realistically simulate the environment in which tumors grow at their site of origin, thus providing a closer approximation of human pathology and biological behavior. Consequently, these experimental findings require further validation and evidence-based studies before clinical implementation. It is crucial to note that biological signaling pathways are often characterized by their intricate complexity and dynamic nature, encompassing numerous protein and gene interactions. While the current investigation focuses on *F. nucleatum*-mediated CDH1 phosphorylation in the context of the CDH1/β-catenin pathway, other associated signaling pathways and molecular regulatory mechanisms could potentially influence the initiation and progression of OSCC. As such, the outcomes of this research warrant further corroboration through clinical investigations to evaluate their prospective utility in the prevention and treatment of OSCC. To address these limitations, additional validation experiments and clinical trials are essential.

Our study confirmed that *F. nucleatum* promotes OSCC cell proliferation via the CDH1/β-catenin pathway. Specifically, *F. nucleatum*-induced phosphorylation of CDH1 led to the upregulation of β-catenin, Myc, and Cyclin D1, and downstream signaling pathways that accelerate cell proliferation. However, noncancerous cells did not exhibit this effect, suggesting that *F. nucleatum*-induced proliferation is specific to cancer cells. Our findings provide new insights into the mechanism by which *F. nucleatum* promotes OSCC progression and highlight the potential of targeting the CDH1/β-catenin pathway as a therapeutic approach.

## Conclusion

In conclusion, our research offers valuable insights into the role of *F. nucleatum* in promoting OSCC cell proliferation, specifically through the modulation of the CDH1/β-catenin pathway. The distinctive effect of *F. nucleatum* on cancer cells compared to noncancerous cells enhances our understanding of the bacterium contribution to OSCC progression. By identifying *F. nucleatum*-induced CDH1 phosphorylation as a key factor in activating the CDH1/β-catenin pathway in OSCC, our study emphasizes the potential of targeting this pathway for therapeutic intervention. Future research should focus on further exploring the molecular mechanisms of *F. nucleatum* in OSCC development and on validating our findings through in vivo models and clinical trials. Ultimately, the knowledge gained from these studies may contribute to the development of novel therapeutic strategies for OSCC prevention and treatment, potentially improving patient outcomes and overall survival rates.

### Electronic supplementary material

Below is the link to the electronic supplementary material.

Supplementary Material 1

Supplementary Material 2

## Data Availability

The data used and/or analysed during the current study available from the corresponding author on reasonable request.
