# Supplementary material for: F. Nucleatum enhances oral squamous cell carcinoma proliferation via E-cadherin/β-Catenin pathway
Source: BMC Oral Health. 2024 May 2;24:518. doi: 10.1186/s12903-024-04252-3 (PMC11064238; doi:10.1186/s12903-024-04252-3)
Supplement: Supplementary file 1 — Supplementary Material 1 [file 12903_2024_4252_MOESM1_ESM.docx]

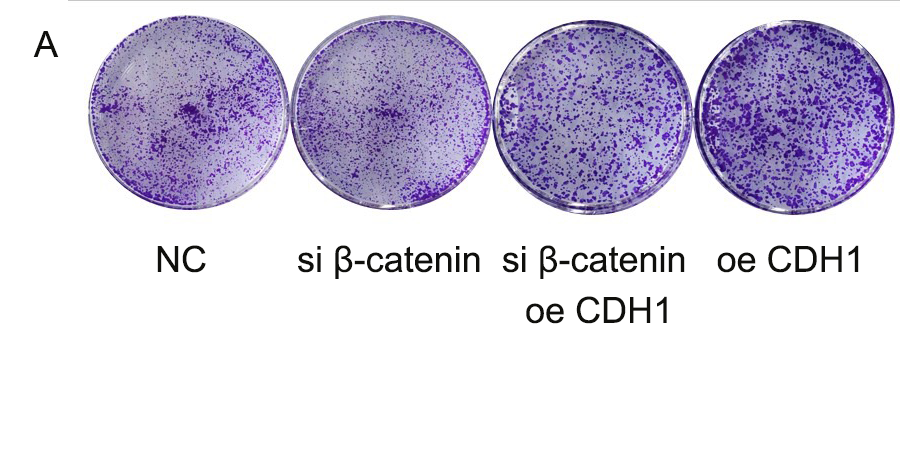


Fig.S1 Detection of *F. nucleatum* on the proliferation of OSCC cells. Compared with the control group, under the same premise of *F. nucleatum* intervention, cell cloning was reduced after knockdown of β-catenin alone. In contrast, cell clones remained unchanged after knockdown of β-catenin and overexpression of CDH1. Cell cloning was significantly increased after overexpression of CDH1 alone.
