# Supplementary material for: F. Nucleatum enhances oral squamous cell carcinoma proliferation via E-cadherin/β-Catenin pathway
Source: BMC Oral Health. 2024 May 2;24:518. doi: 10.1186/s12903-024-04252-3 (PMC11064238; doi:10.1186/s12903-024-04252-3)

Fig. 2 A. Western blot (WB) was employed to monitor the modulation of CDH1 and its downstream proteins after F. nucleatum exposure.

CDH1


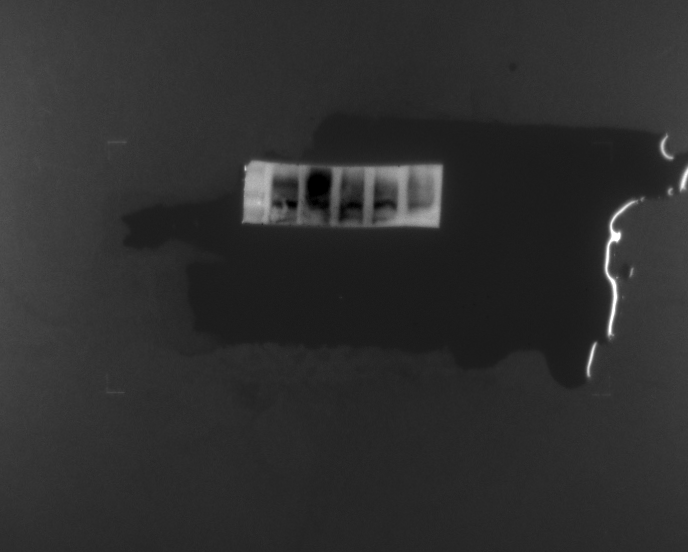

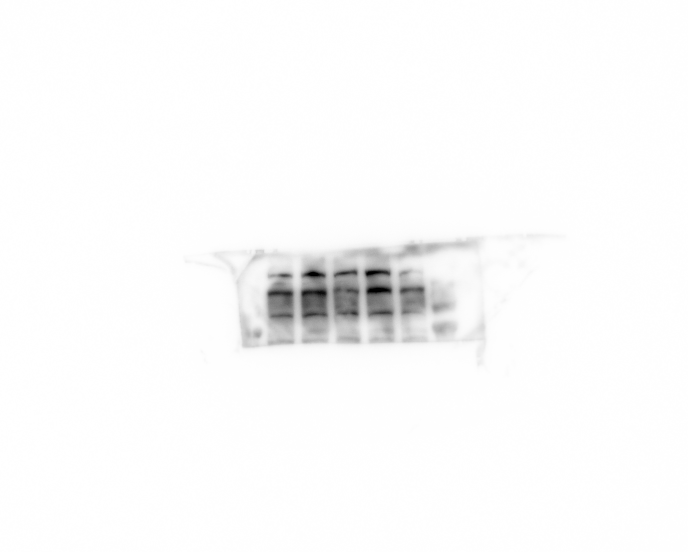


p-CDH1


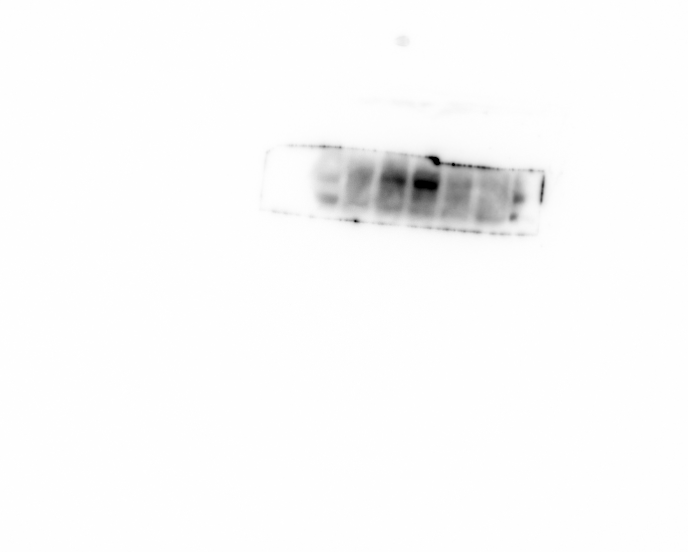

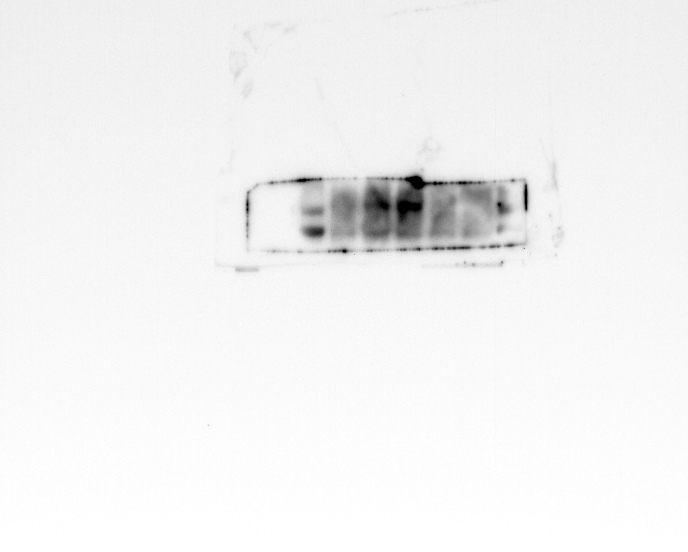

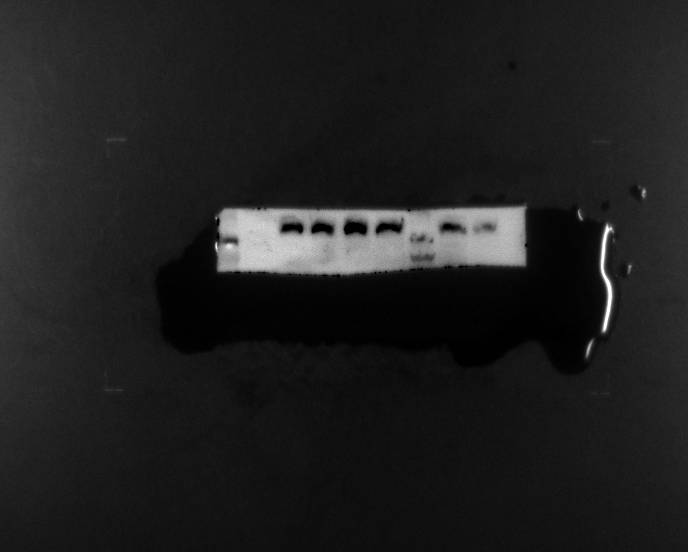


p-CDH1


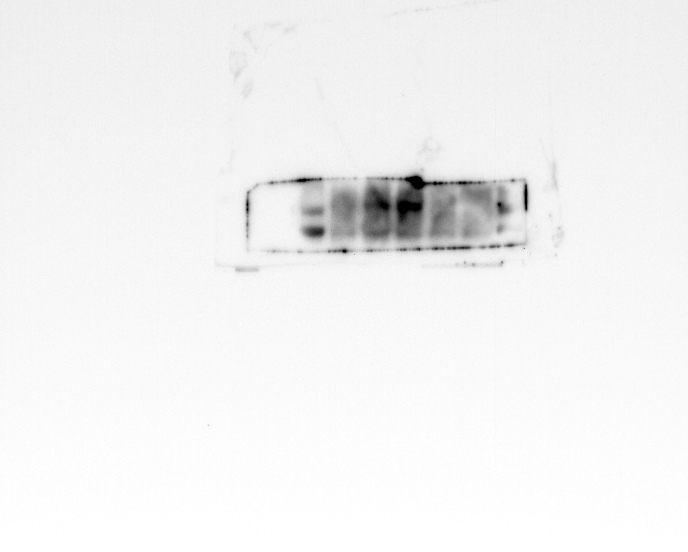

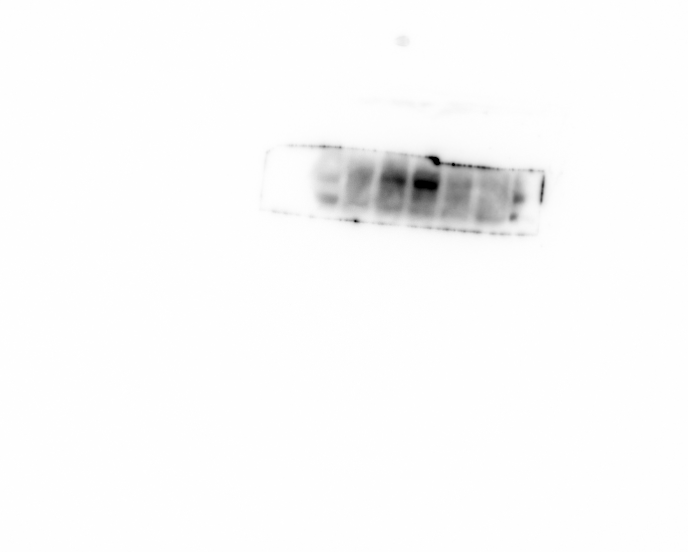

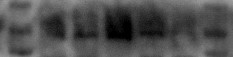


β-catenin


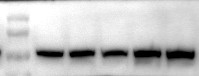

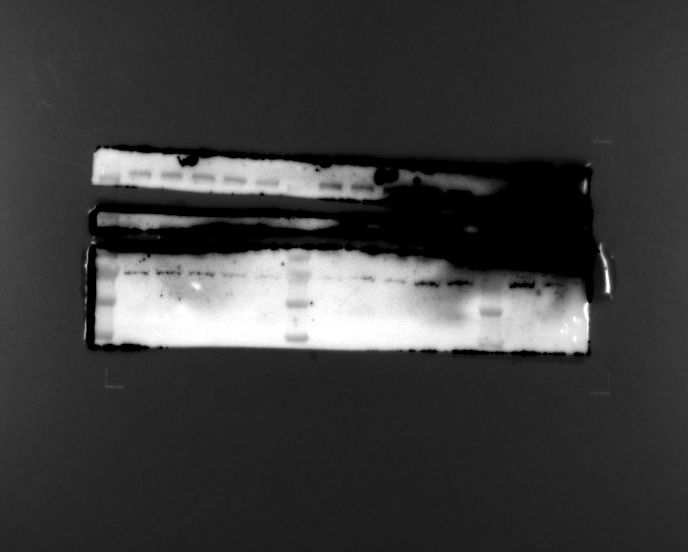

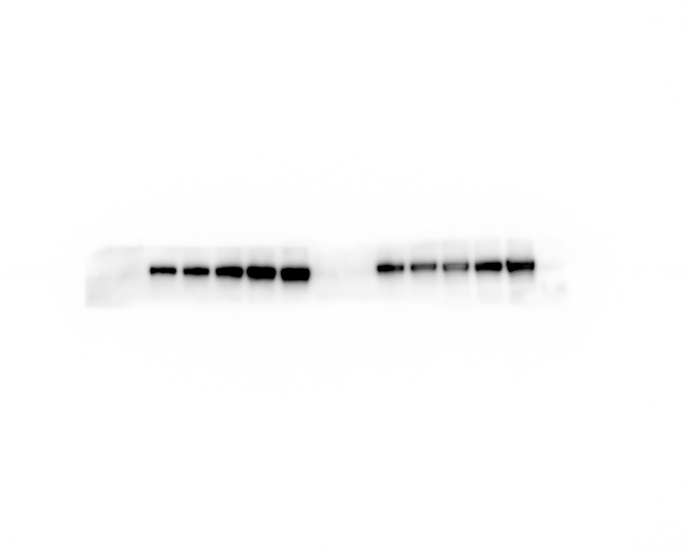


Myc


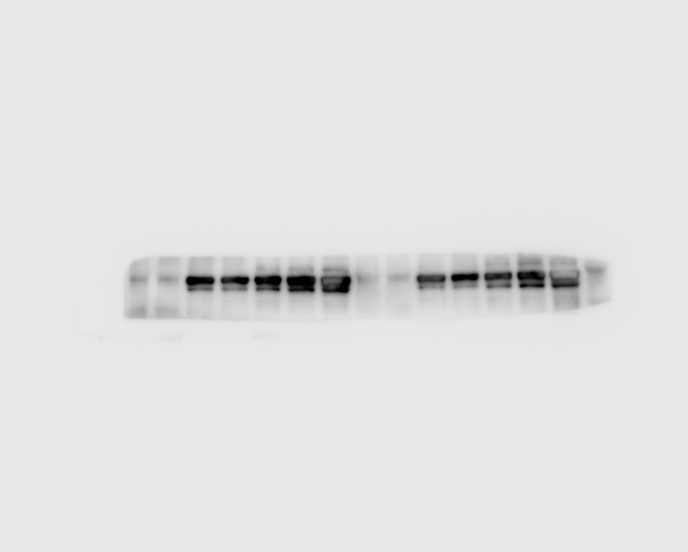

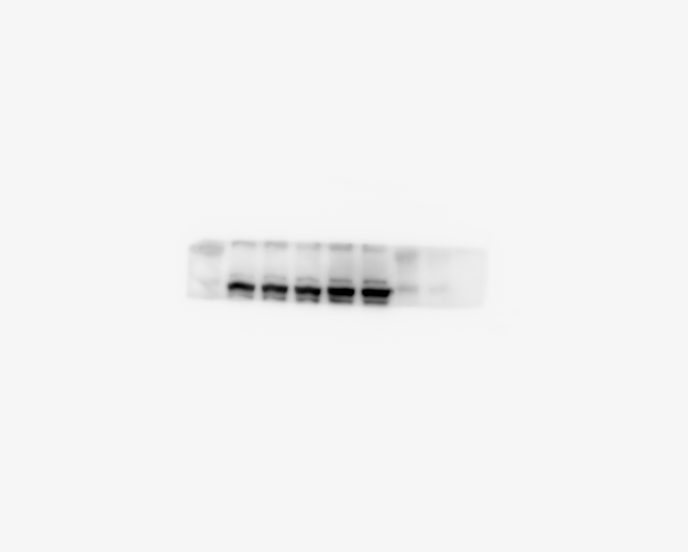


Cyclin D1


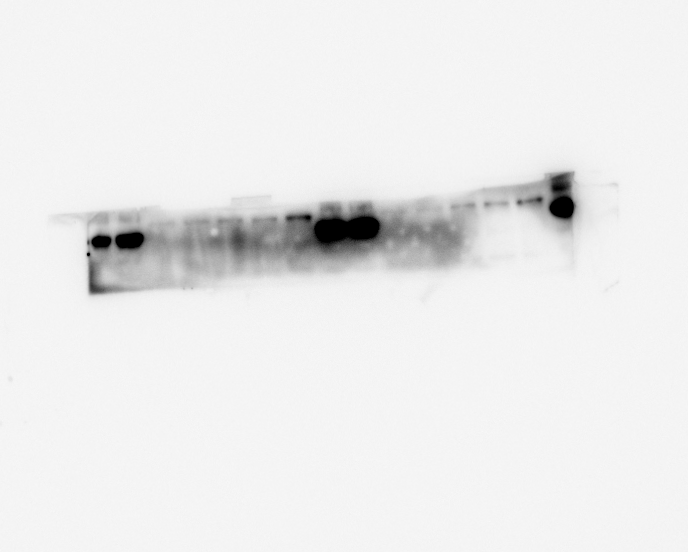

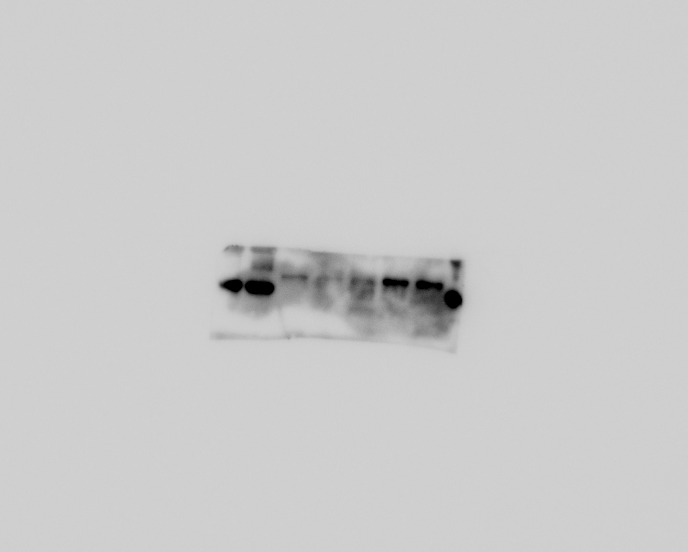

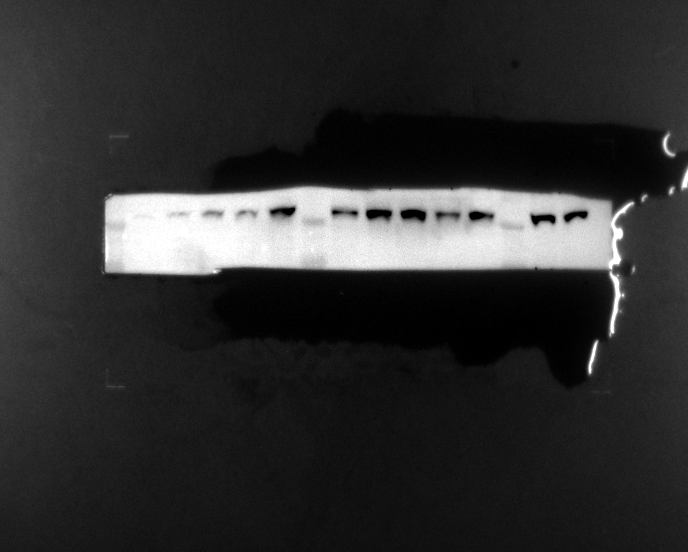


B. Post-genistein treatment analysis revealed an inhibition in CDH1 phosphorylation, ensuring stable expression of CDH1 and its downstream entities.
CDH1


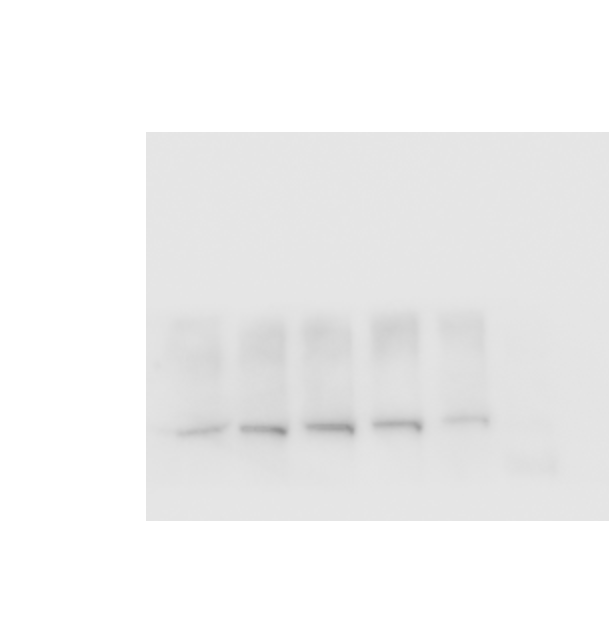

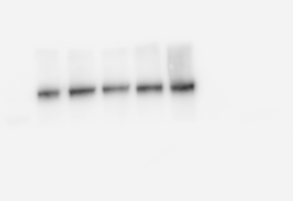

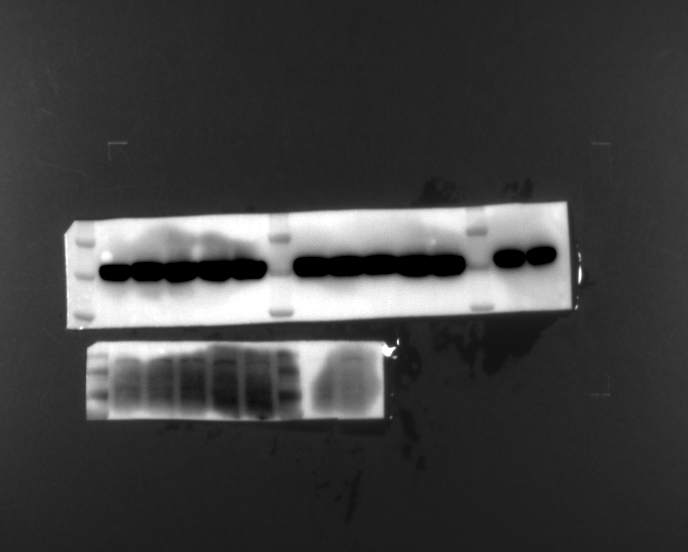


Myc


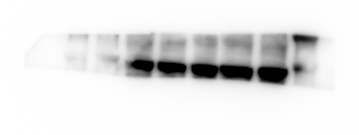

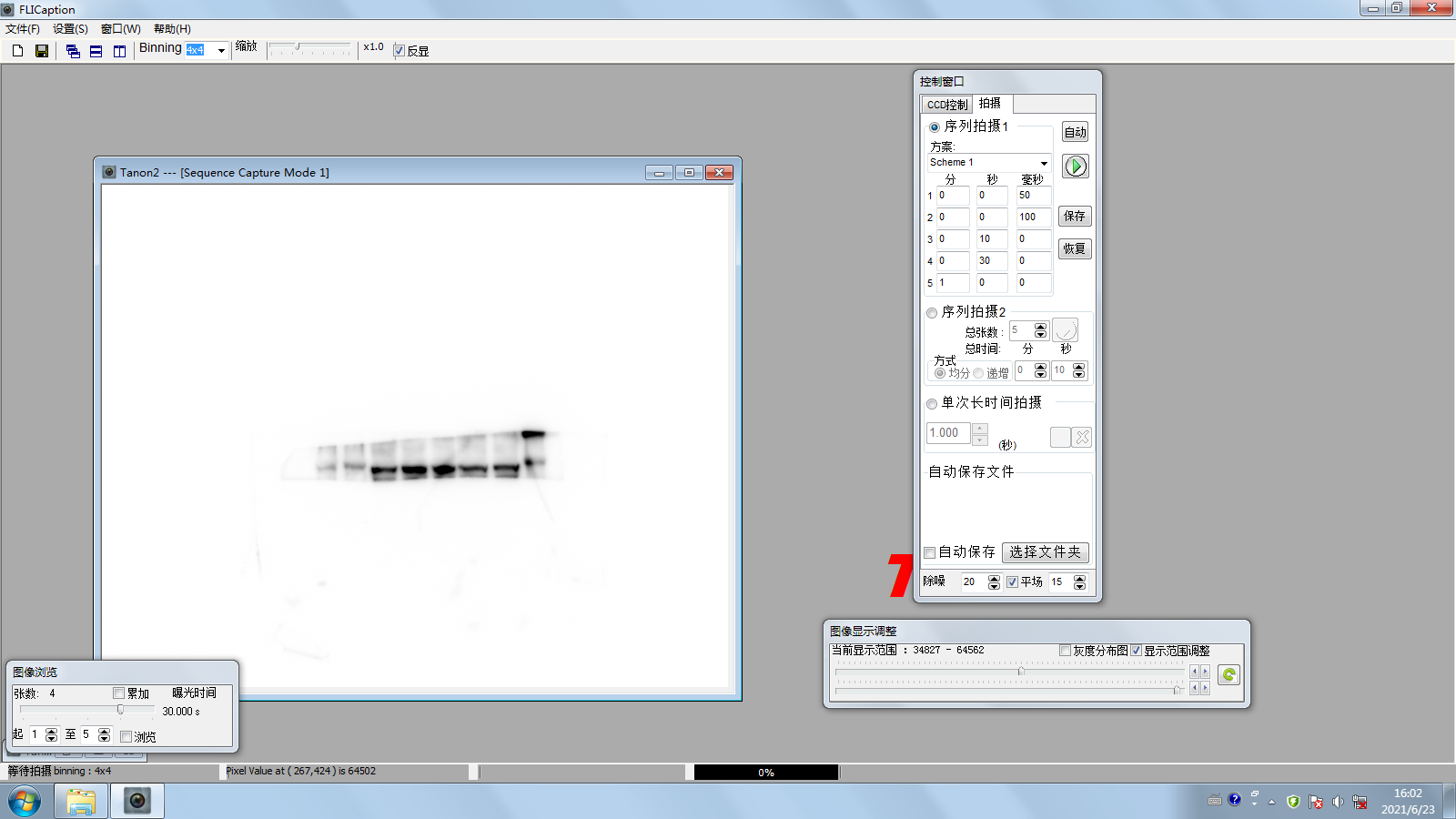

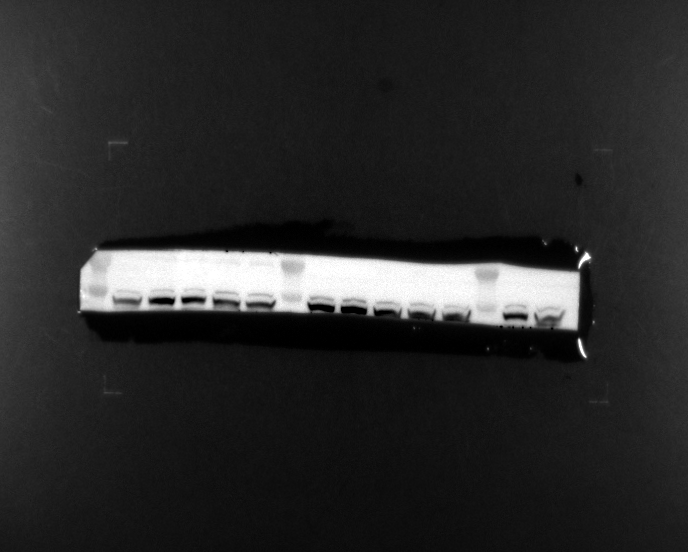


Cyclin D1


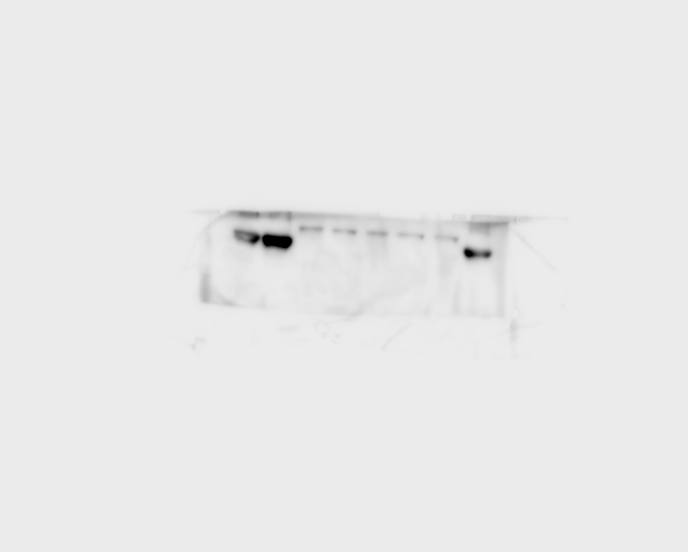

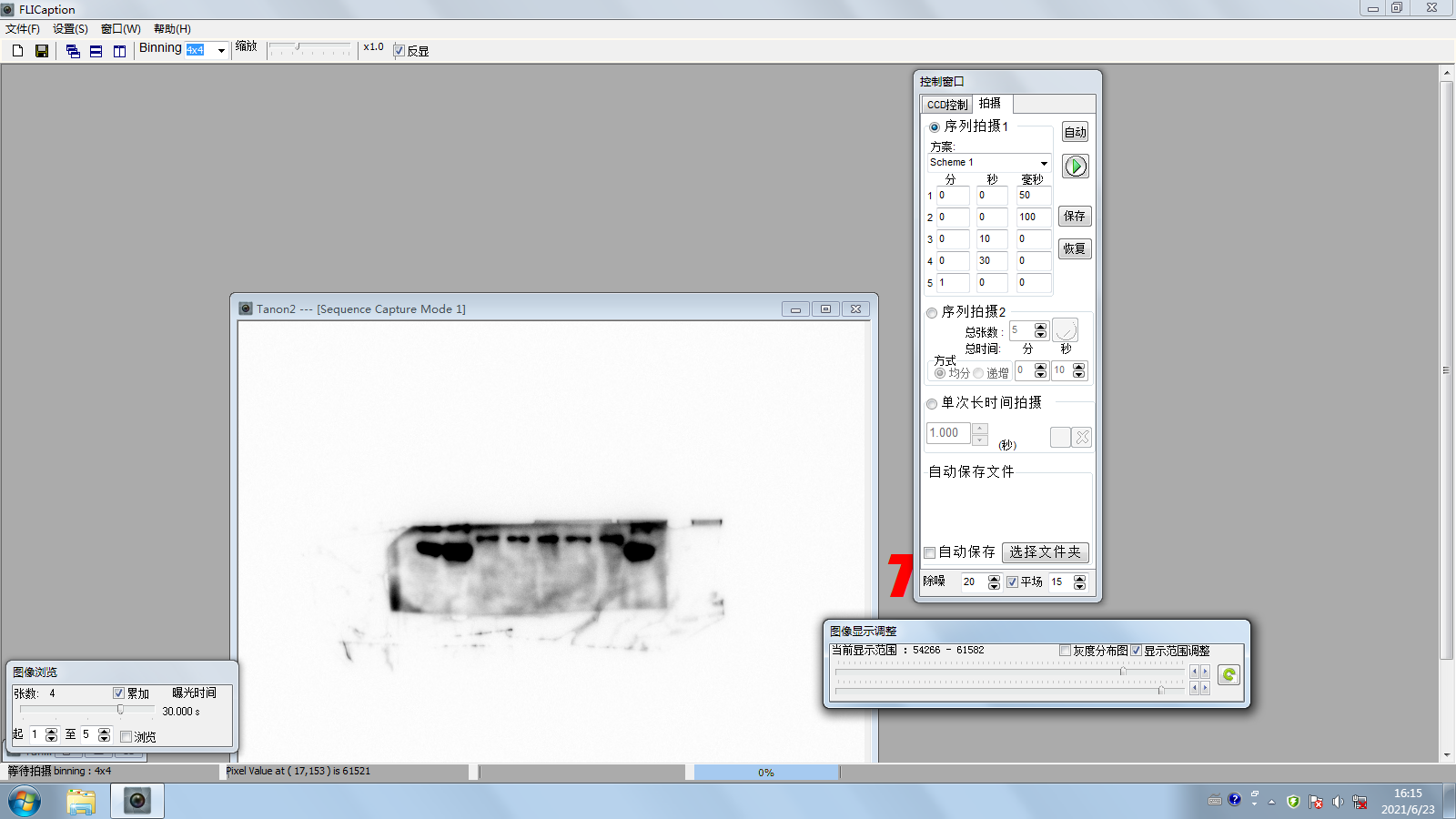

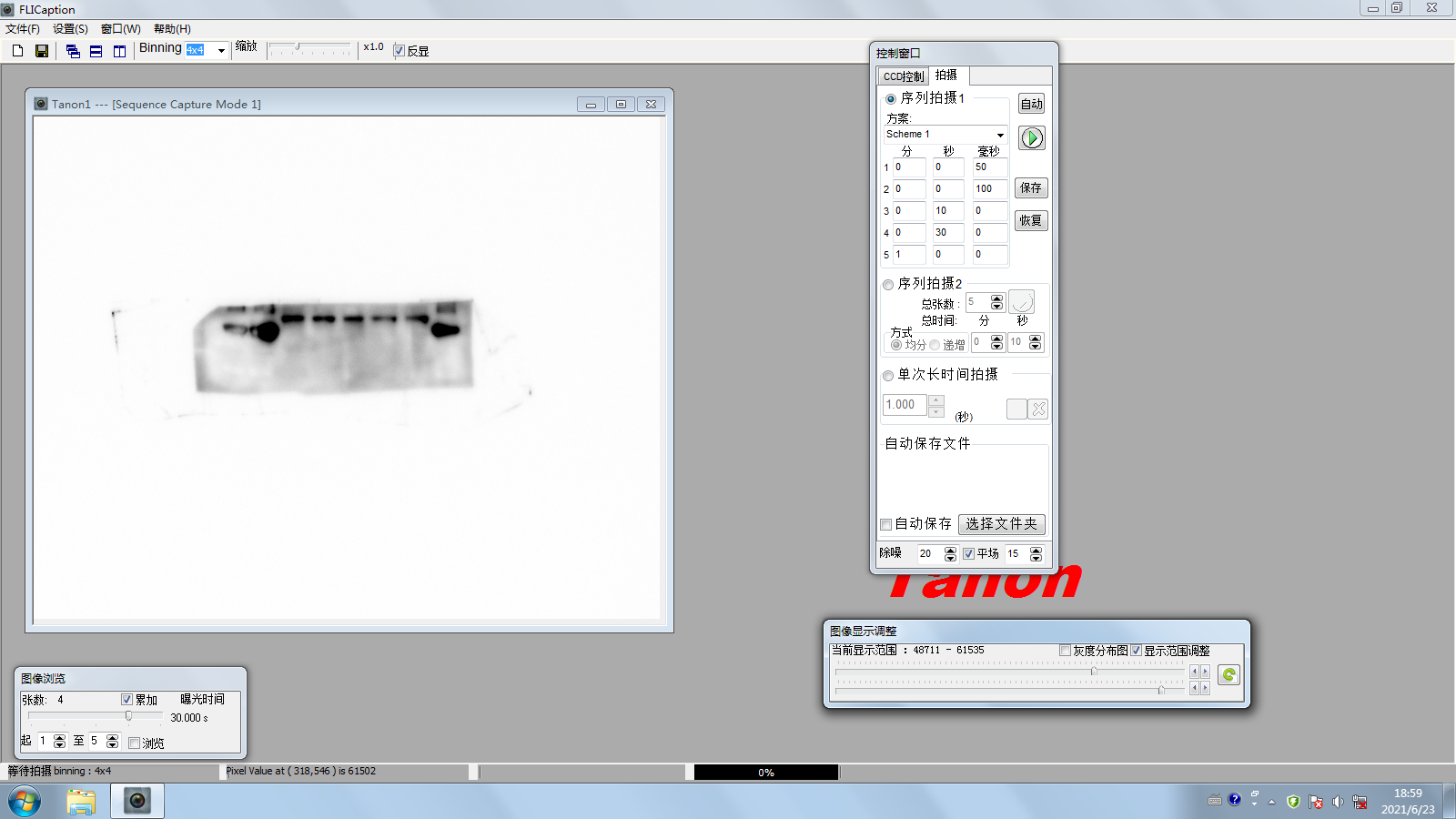

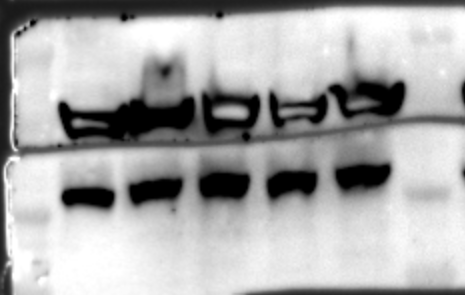


β-catenin


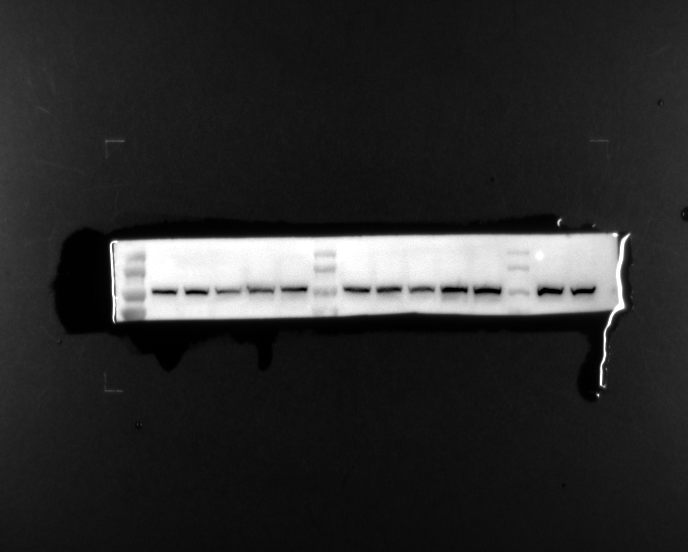

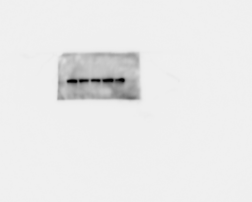


Fig. 3 A. Changes in the expression of CDH1 and its downstream proteins after transient transfection. After knocking down β-catenin, downstream protein expression was inhibited. After CDH1 overexpression, oncogene expression increased slightly.

CDH1


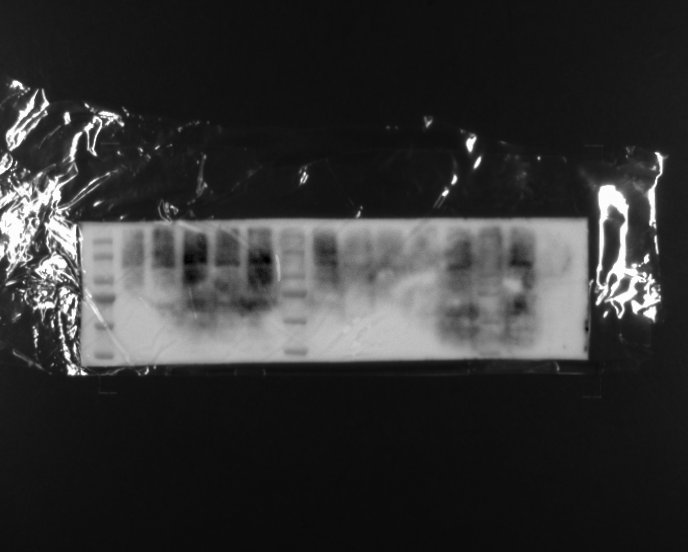







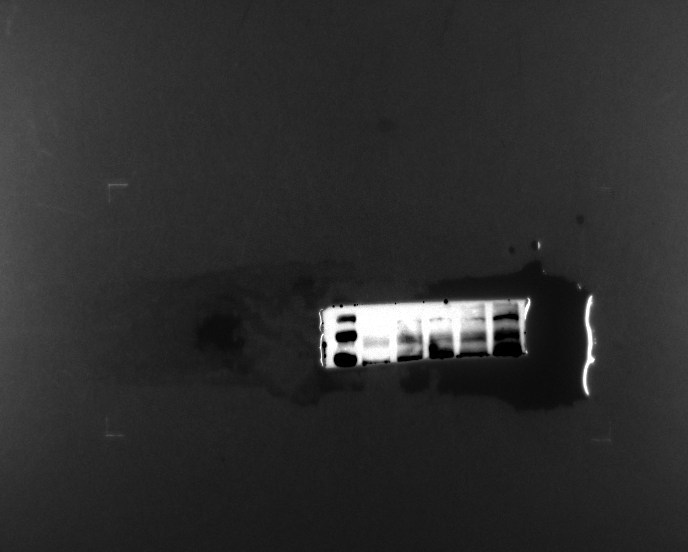


p-CDH1


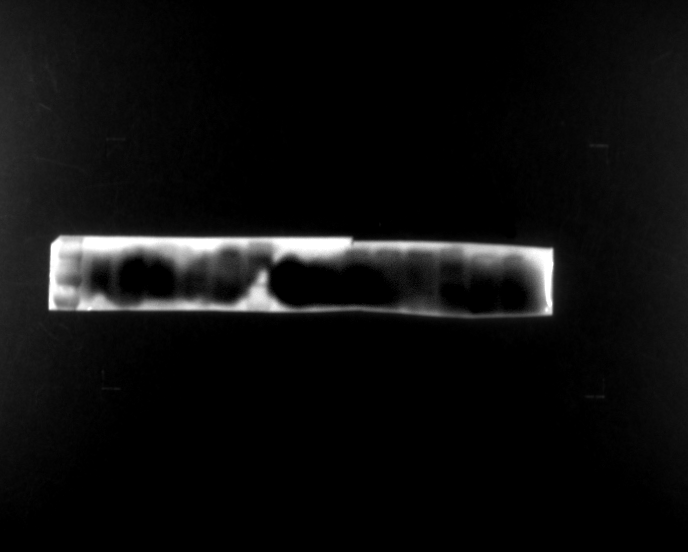

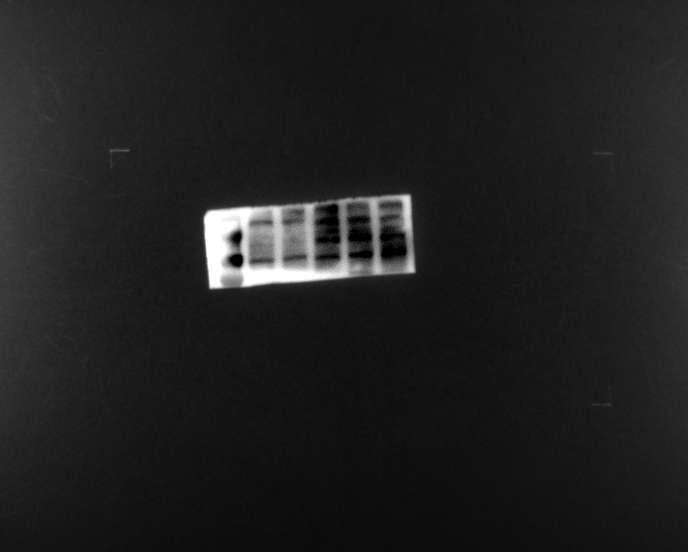
.
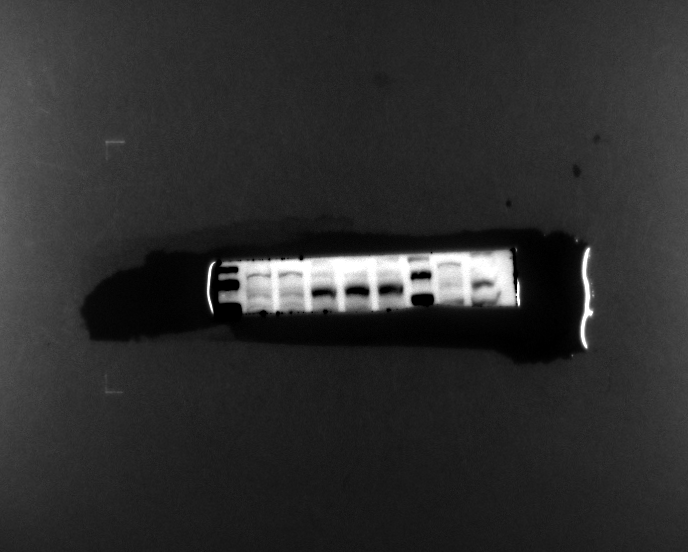


β-catenin


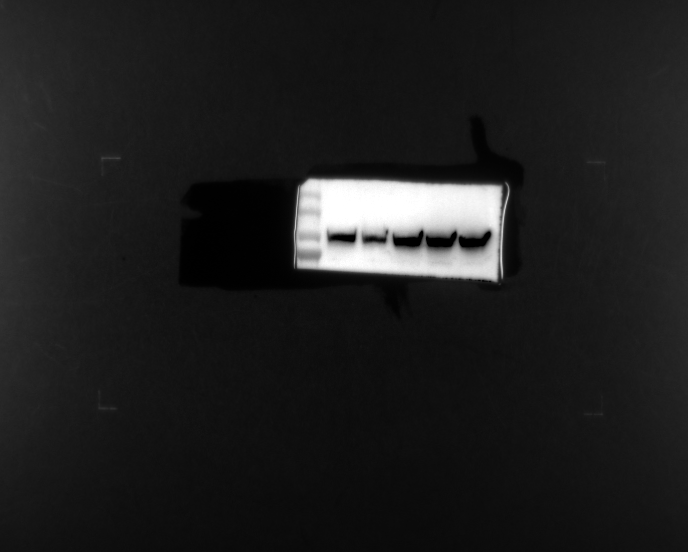

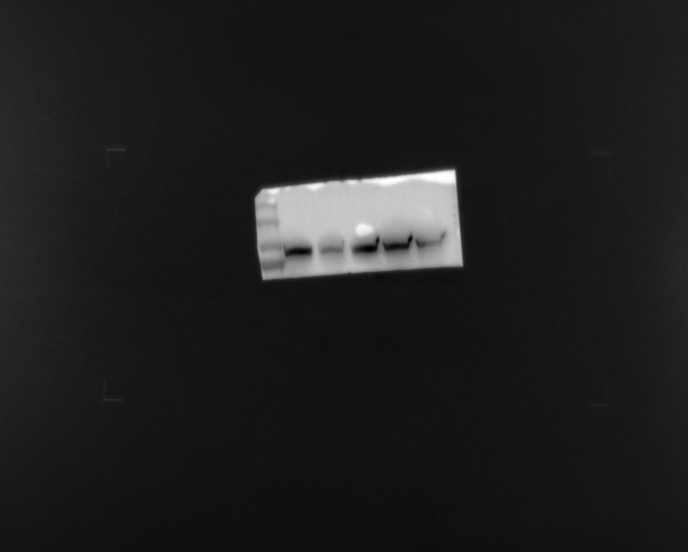


Myc









Cyclin D1









Fig. 4 A. Detection of CDH1 expression in four cell lines revealed differential expression in tumor cell lines, human oral keratinocytes (HOK), 293T cells, and human gingival fibroblasts(HGF).


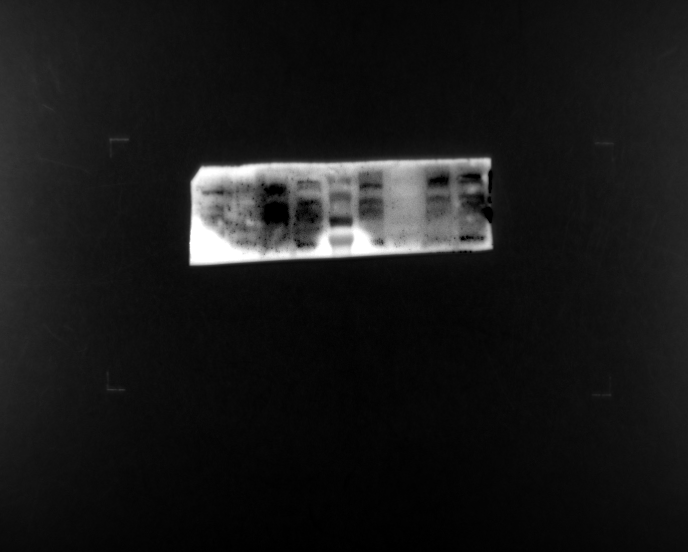

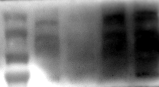


Fig. 4 D. In 293T cells overexpressing CDH1, phosphorylated CDH1 was detected, but downstream signaling was not activated.

CDH1


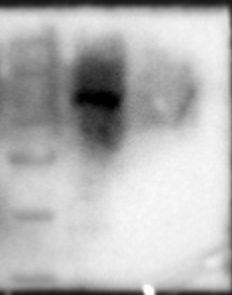

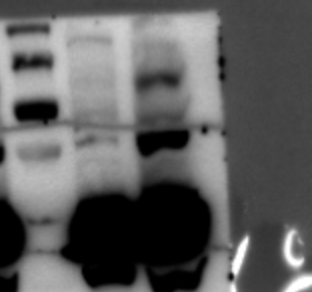


p-CDH1


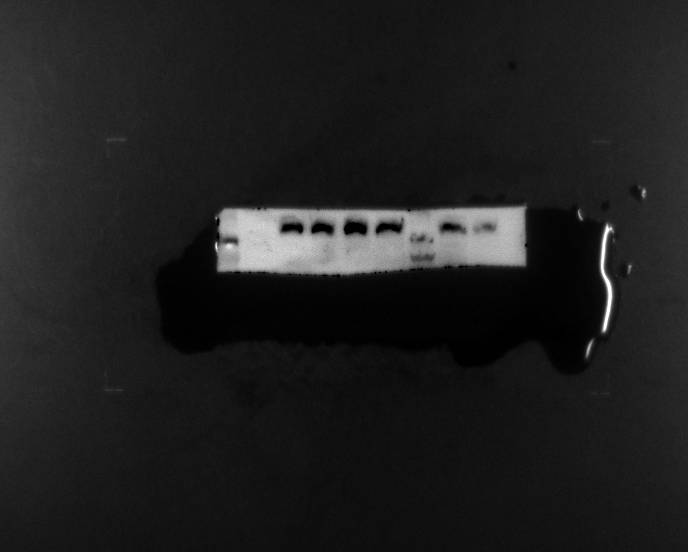

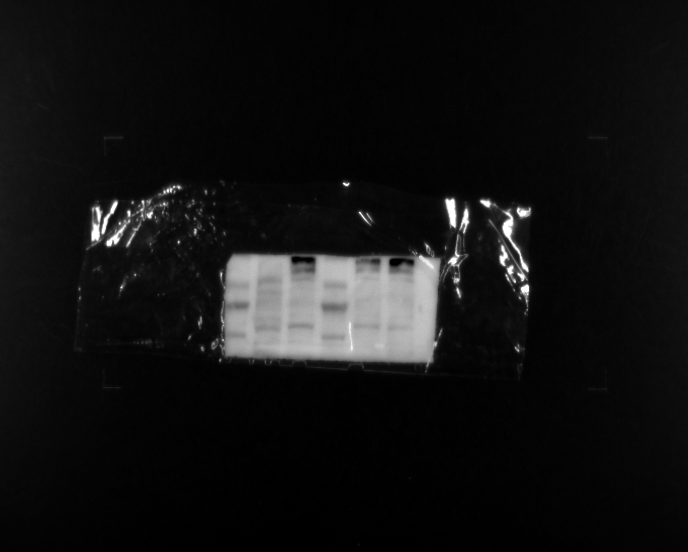


β-catenin


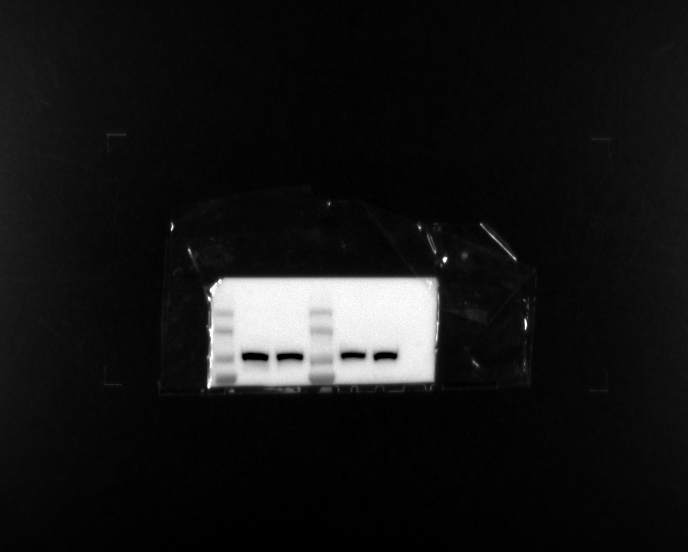

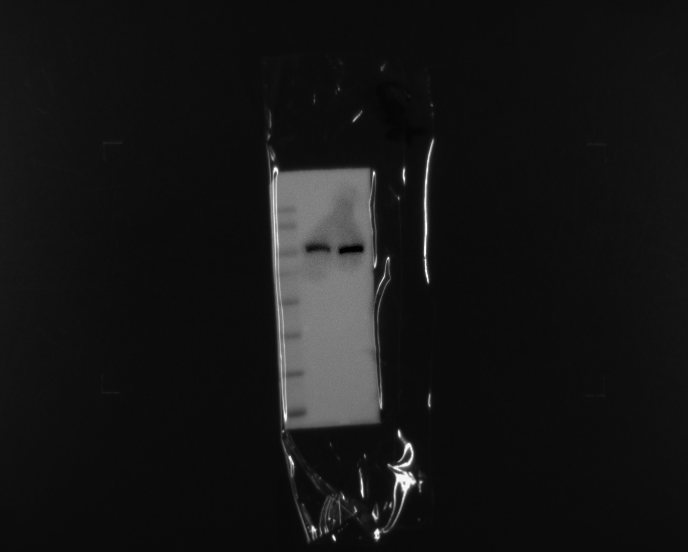




Myc


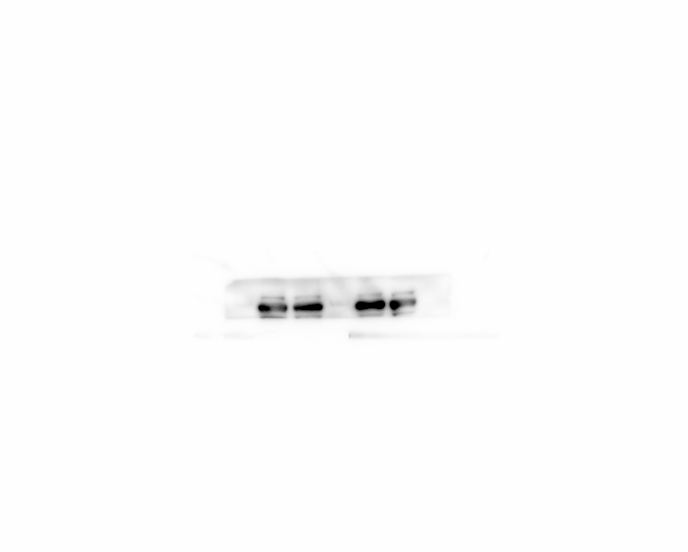

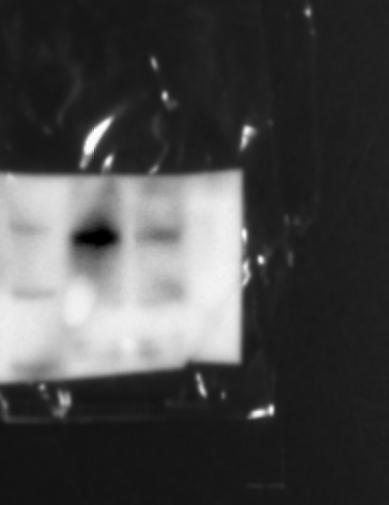


Cyclin D1


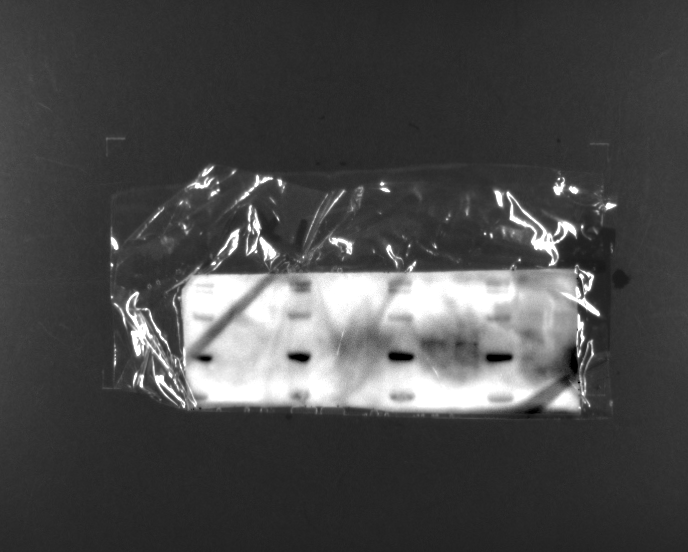

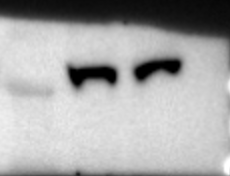

Supplement: Supplementary file 2 — Supplementary Material 2 [file 12903_2024_4252_MOESM2_ESM.docx]
